# Task force of the Brazilian Society of Otology — evaluation and management of peripheral facial palsy

**DOI:** 10.1016/j.bjorl.2023.101374

**Published:** 2023-12-08

**Authors:** Henrique Furlan Pauna, Vagner Antonio Rodrigues Silva, Joel Lavinsky, Miguel Angelo Hyppolito, Melissa Ferreira Vianna, Mariana de Carvalho Leal Gouveia, Rafael da Costa Monsanto, José Fernando Polanski, Maurício Noschang Lopes da Silva, Vítor Yamashiro Rocha Soares, André Luiz Lopes Sampaio, Raul Vitor Rossi Zanini, Nicolau M. Abrahão, Guilherme Correa Guimarães, Carlos Takahiro Chone, Arthur Menino Castilho

**Affiliations:** a Hospital Universitário Cajuru, Departamento de Otorrinolaringologia, Curitiba, PR, Brazil; b Universidade Estadual de Campinas (UNICAMP), Departamento de Otorrinolaringologia e Cirurgia de Cabeça e Pescoço, Campinas, SP, Brazil; c Universidade Federal do Rio Grande do Sul (UFRGS), Departamento de Cirurgia, Porto Alegre, RS, Brazil; d Universidade de São Paulo (USP), Faculdade de Medicina de Ribeirão Preto, Departamento de Oftalmologia, Otorrinolaringologia e Cirurgia de Cabeça e Pescoço, Ribeirão Preto, SP, Brazil; e Irmandade Santa Casa de Misericórdia de São Paulo, Departamento de Otorrinolaringologia, São Paulo, SP, Brazil; f Universidade Federal de Pernambuco (UFPE), Departamento de Cirurgia, Recife, PE, Brazil; g University of Minnesota, Department of Otolaryngology, Head & Neck Surgery, Minneapolis, USA; h Universidade Federal do Paraná (UFPR), Hospital de Clínicas, Departamento de Otorrinolaringologia e Cirurgia de Cabeça e Pescoço, Curitiba, PR, Brazil; i Hospital de Clínicas de Porto Alegre (UFRGS), Departamento de Otorrinolaringologia e Cirurgia de Cabeça e Pescoço, Porto Alegre, RS, Brazil; j Hospital Flávio Santos and Hospital Getúlio Vargas, Grupo de Otologia e Base Lateral do Crânio, Teresina, PI, Brazil; k Universidade de Brasília (UnB), Faculdade de Medicina, Laboratório de Ensino e Pesquisa em Otorrinolaringologia, Brasília, DF, Brazil; l Hospital Israelita Albert Einstein, Departamento de Otorrinolaringologia, São Paulo, SP, Brazil

**Keywords:** Facial palsy, Facial paralysis, Bell palsy, Facial nerve disease, Herpes Zoster *Oticus*, Facial nerve trauma, Microvascular decompression surgery, Guidelines

## Abstract

**Objective:**

To review key evidence-based recommendations for the diagnosis and treatment of peripheral facial palsy in children and adults.

**Methods:**

Task force members were educated on knowledge synthesis methods, including electronic database search, review and selection of relevant citations, and critical appraisal of selected studies. Articles written in English or Portuguese on peripheral facial palsy were eligible for inclusion. The American College of Physicians’ guideline grading system and the American Thyroid Association’s guideline criteria were used for critical appraisal of evidence and recommendations for therapeutic interventions.

**Results:**

The topics were divided into 2 main parts: (1) Evaluation and diagnosis of facial palsy: electrophysiologic tests, idiopathic facial palsy, Ramsay Hunt syndrome, traumatic peripheral facial palsy, recurrent peripheral facial palsy, facial nerve tumors, and peripheral facial palsy in children; and (2) Rehabilitation procedures: surgical decompression of the facial nerve, facial nerve grafting, surgical treatment of long-term peripheral facial palsy, and non-surgical rehabilitation of the facial nerve.

**Conclusions:**

Peripheral facial palsy is a condition of diverse etiology. Treatment should be individualized according to the cause of facial nerve dysfunction, but the literature presents better evidence-based recommendations for systemic corticosteroid therapy.

## Highlights

• This is an evidence-based review on the recommendations for the diagnosis and treatment of Peripheral Facial Palsy (PFP).
• The diagnosis of PFP is based on epidemiological data, medical history, and physical examination.
• As the nerve regenerate, neural desynchronization and muscle depolarization occurs at different intervals.
• Surgical decompression of the facial nerve is suggested when electrophysiologic testing shows degeneration greater than 90%.

## Introduction

Peripheral Facial Palsy (PFP) refers to a lower motor neuron lesion of the seventh Cranial Nerve (CN VII) and can occur in any part of the distal segment of the facial nerve. It results from several medical conditions, such as infection, cholesteatoma, trauma, malignancy, autoimmune disorders, and pregnancy.^1^ Although the idiopathic form (Bell’s palsy) is the most common, viral reactivation (Herpes Simplex Virus [HSV] type 1 or Varicella Zoster Virus [VZV]) is assumed in a considerable number of PFP cases.^1^

Regarding terminology, “paresis” is often used to describe incomplete CN VII injury, whereas “paralysis” (or “palsy” in combination) is used if the injury is complete. As for laterality, PFP occurs unilaterally in most cases, but bilateral PFP is also possible, although rare.^1^

The incidence of idiopathic PFP is estimated at 20–30 cases per 100,000 population, being slightly more common in women.^2^ In children, PFP has an overall incidence of 2.7 per 100,000 children under the age of 10, and 10.1 per 100,000 children over the age of 10 annually.^3^

The diagnosis of PFP in adults and children is based on local epidemiological data, medical history, and physical examination. The three-thirds of the face should be assessed to grade mobility and asymmetry at rest and during motion, applying the available scales (e.g., House-Brackmann [HB], Fisch, or Sunnybrook grading systems).^3^, ^4^ Imaging should be performed in cases with unfavorable outcome, without recovery within two months, recurrence and worsening progression, and for evaluation of the finding of a tumor mass during physical examination.^3^, ^5^ Whenever performed, the imaging study must include images of the brain, cerebellopontine angle, Internal Auditory Canal (IAC), facial nerve canal (Fallopian canal), geniculate ganglion, stylomastoid foramen, and parotid glands.^5^

The need to establish the prognosis and outcome of PFP has led to the development of different methods to quantify the facial nerve involvement clinically. Objective and subjective methods have been developed for this assessment, based on the presence or absence of pre-established facial movements.^2^ The most used method is the HB grading system, developed in 1985, but other scales have also been suggested (Table 1, Table 2, Table 3, Table 4, Table 5; Appendices 1–3).^2^, ^6^, ^7^, ^8^, ^9^, ^10^, ^11^

**Table 1:** Assessment of facial movement according to House-Brackmann (HOUSE & BRACKMANN, 1985).

Grade	Description	At rest	Motion
I	Normal	Symmetry	Normal facial function
II	Mild dysfunction	Normal symmetry and tone	Forehead: moderate to good function
			Eye: complete closure with minimum effort
			Mouth: slight asymmetry
III	Moderate dysfunction	Normal symmetry and tone	Forehead: slight to moderate movement
			Eye: complete closure with minimum effort
			Mouth: slightly weak with maximum effort
IV	Moderately severe dysfunction	Normal symmetry and tone	Forehead: none
			Eye: incomplete closure
			Mouth: asymmetric with maximum effort
V	Severe dysfunction	Asymmetry	Eye: incomplete closure
			Mouth: slight movement
VI	Total paralysis	Asymmetry	No movement

**Table 2:** Assessment of facial movement according to Chevalier [FONSECA et al., 2015].

Level	Description
0	Contraction not visible to the naked eye nor with oblique light incidence
1	Slight mobility of skin
2	The skin has more mobility. Wrinkles are lightly perceived
3	The skin moves more clearly. The number of wrinkles increases, as well as their depth
4	The movement takes place in a wide, synchronous, and symmetrical manner in relation to the uninjured side

**Table 3:** Assessment of facial movement according Yanagihara (KIM et al., 2013).

Movement	No mobility		Moderate	Normal	
At rest	0	1	2	3	4
Wrinkle forehead	0	1	2	3	4
Blink	0	1	2	3	4
Closure of eye lightly	0	1	2	3	4
Closure of eye tightly	0	1	2	3	4
Closure of eye on the involved side only	0	1	2	3	4
Wrinkle nose	0	1	2	3	4
Whistle	0	1	2	3	4
Grin	0	1	2	3	4
Depress lower lip	0	1	2	3	4

**Table 4:** Assessment of facial movement according to the Sunnybrook scale (NEELY et al., 2010).

At rest (compared with normal side)
Eyes (choose one)
- Normal (0)
- Eyelids expose the iris more than the other side [wide 1]
- Eyelids expose the iris less than on the other side, as if squinting [narrow 1]

Nasolabial fold (choose one)
-Normal (0)
- Fold less pronounced than on the other side [less evident 1]
- Fold not visible [absent 2]
- Fold more pronounced than on the other side [more evident 1]

Mouth (choose one)
- Normal (0)
- Corner more dropped than on the other side [corner dropped 1]
- Corner more pulled up than on the other side [corner pulled up 1]

Voluntary movement (compared with normal side)
Brow lift: lifting the brows for wrinkling forehead
- Normal (5)
- The forehead wrinkles well; hard to see the difference [almost normal 4]
- Obvious movement, but not almost normal [moderate 3]
- The forehead barely moves; hard to see movement [mild 2]
- No forehead movement [none 1]

Gentle eye closure
- Eyelids close completely and at the same speed [normal 5]
- Eyelids close completely but at slower speed [almost normal 4]
- Eyelids do not close completely, leaving only a narrow slit of the eyeball exposed [moderate 3]
- Eyelids do not close completely; half closed [mild 2]
- Eyelids do not close; more than half of eyeball exposed [none 1]

Snarl Elevating the center of the face as if smelling something bad (choose one)
- Normal (5)
- Almost identical to the other side; difficult to see difference [almost normal 4]
- Obvious movement, but not almost normal [moderate 3]
- Barely moves; hard to see movement [mild 2]
- No movement [none 1]

Open mouth smile
- Normal (5)
- Almost identical to the other side; difficult to see difference [almost normal 4]
- Obvious movement, but not almost normal [moderate 3]
- Barely moves; hard to see movement [mild 2]
- No movement [none 1]

Lip pucker: lips pursed as if to whistle. Look at the affected side and compare it with the normal one (choose one)
- Normal (5)
- Almost uniformly symmetrical; difficult to see difference [almost normal 4]
- Obviously asymmetrical; lip protrusion on the affected side [moderate 3]
- Mild flat movement of the labial commissure of mouth, but no protrusion [mild 2]
- No movement [none 1]
Synkinesis: involuntary muscle contraction greater than on the normal side in a region distant from the region of the requested movement. Compare with the normal side.

Requested voluntary movement
Involuntary synkinetic movement (greater than on the normal side)
Brow lift
Eyes and/or mouth
- None (0)
- Slight, only visible if looking close (1)
- Moderate, easy to see (2)
- Severe, grossly disfiguring (rare 3)
Gentle eye closure
Brow and/or mouth
- None (0)
- Slight, only visible if looking close (1)
- Moderate, easy to see (2)
- Severe, extremely disfiguring (rare 3)
Snarl
Brow, eyes, and/or mouth
- None (0)
- Slight, only visible if looking close (1)
- Moderate, easy to see (2)
- Severe, grossly disfiguring (rare 3)
Open mouth smile
Brow and/or mouth
- None (0)
- Slight, only visible if looking close (1)
- Moderate, easy to see (2)
- Severe, extremely disfiguring (rare 3)
Lip pucker
Brow and/or eyes
- None (0)
- Slight, only visible if looking close (1)
- Moderate, easy to see (2)
- Severe, grossly disfiguring (rare 3)

**Table 5:** Assessment of facial movement according to Fisch [1981].

Symmetry of face	%	Points
At rest	0	0
	30	6
	70	14
	100	20
Wrinkling forehead	0	0
	30	3
	70	7
	100	10
Closing eyes	0	0
	30	9
	70	21
	100	30
Smiling	0	0
	30	9
	70	21
	100	30
Whistling	0	0
	30	3
	70	7
	100	10

For each of five positions of face, evaluator had four choices. A total of 100 points was divided between different positions of face according to following key: face at rest, 20 points; wrinkling forehead, 10; closing eyes, 30; smiling, 30; whistling, 10. Total points can be used to express facial symmetry in percent.

## The facial nerve

### Embryology

The acoustic-facial placode, which will give rise to the vestibulocochlear and facial nerves, appears during the third week of life. The formation of the facial muscles begins at four weeks’ gestation and, at week five, the main branches of the facial nerve can be identified, which, in association with the facial muscles, will migrate from lateral to medial to form the face. Extensive branching of the facial nerve occurs between 10 and 15 weeks. The facial nerve canal (Fallopian canal) surrounds the facial nerve in its intratemporal segment from 10 weeks’ gestation onward, but its ossification continues until years after birth.^12^

### Histology

The facial nerve is a mixed nerve consisting of approximately 10,000 fibers, 80% motor fibers and 20% special sensory fibers [VIANNA, 2009].^12^ The fibers (axons) that make up the facial nerve are surrounded by the myelin sheath. This sheath consists of multiple layers of lipid-rich cytoplasm that confer characteristics of electrical insulation, allowing saltatory conduction through the nodes of Ranvier, which increases the conduction velocity of the electrical stimulus from 0.2 to 2 m/s (unmyelinated fibers) to 5–100 m/s (myelinated fibers).^12^, ^13^

**Figure 1:**
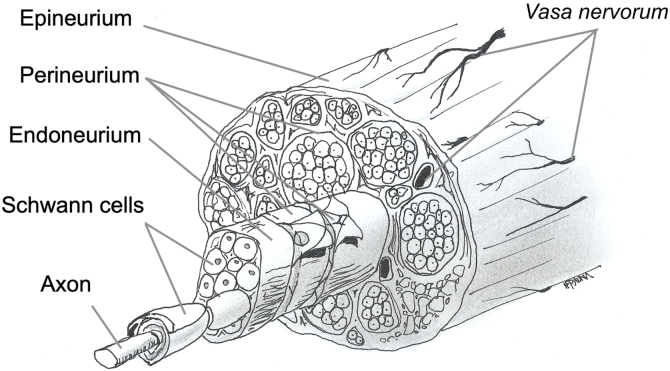
Schematic representation of nerve structure.

Nerve fibers are embedded in loose connective tissue called the endoneurium. Each group of fibers is surrounded by the perineurium, forming a fascicle, and these fascicles are embedded in dense connective tissue called the epineurium (Fig. 1).

The facial nerve has a variable number of fascicles, with only one in the proximal segment and an increasing number in the intratemporal segment, where it divides into several branches. The number of fibers decreases toward the extratemporal segment. Knowledge of the neural structure allows understanding the types of nerve injury and the likelihood of recovery and resulting sequelae.^12^, ^13^

### Anatomy

The facial nerve forms in the facial nuclei in the central nervous system, in the pons. On each side, it is possible to identify a superior nucleus and an inferior nucleus. The fibers arriving in the nuclei come from the cerebral cortex, from the motor area of the precentral gyrus, through the corticonuclear pathway, and receive their blood supply from the middle cerebral artery. Most fibers from the cerebral cortex will synapse in the contralateral facial nucleus, but a portion of these fibers will synapse in the ipsilateral superior nucleus. In the pons, the facial nuclei are supplied by the anterior inferior cerebellar artery.^14^

**Figure 2:**
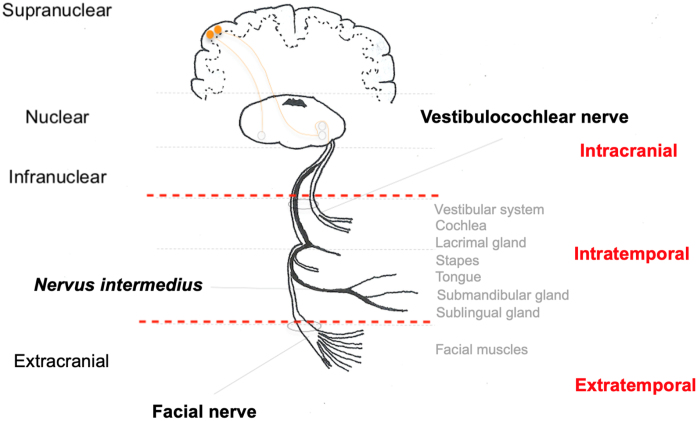
Anatomical schematic representation of the facial nerve divided into segments and intracranial and extracranial innervated structures.

The fibers forming the facial nerve emerge from the facial nuclei in the pons. Also in the pons, facial nerve fibers surround the nucleus of the abducens nerve (CN VI) and exit the central nervous system into the region of the cerebellopontine angle cistern. The nerve segment extending from the exit of the pons to the entrance of the IAC is called the cisternal segment. Then, the nerve travels along the temporal bone, inside the Fallopian canal, until it reaches the facial muscles in its extratemporal segment (Fig. 2).^14^

In the cisternal segment, the facial nerve is not covered by an epineurium and is surrounded by the pia mater. A layer of dura mater surrounds both the facial nerve and the vestibulocochlear nerve (CN VIII). Both in the cisternal segment and the IAC, the facial nerve is located anterior to and superior to the vestibulocochlear nerve.^15^

Intratemporally, the facial nerve runs a tortuous course through its bony canal and is divided into three segments: labyrinthine segment, which runs from the IAC to the first genu, where the geniculate ganglion is located; tympanic or horizontal segment, between the first and second genu of the facial nerve; and mastoid or vertical segment, from the second genu to the stylomastoid foramen, where the facial nerve exits the temporal bone toward the facial muscles, where it is then called the extratemporal facial nerve. In its course, the facial nerve connects with the nervus intermedius, which has sensory and parasympathetic fibers that will provide innervation to the lacrimal, submandibular, and sublingual glands and taste to the anterior two-thirds of the tongue and part of the palate.^15^

**Figure 3:**
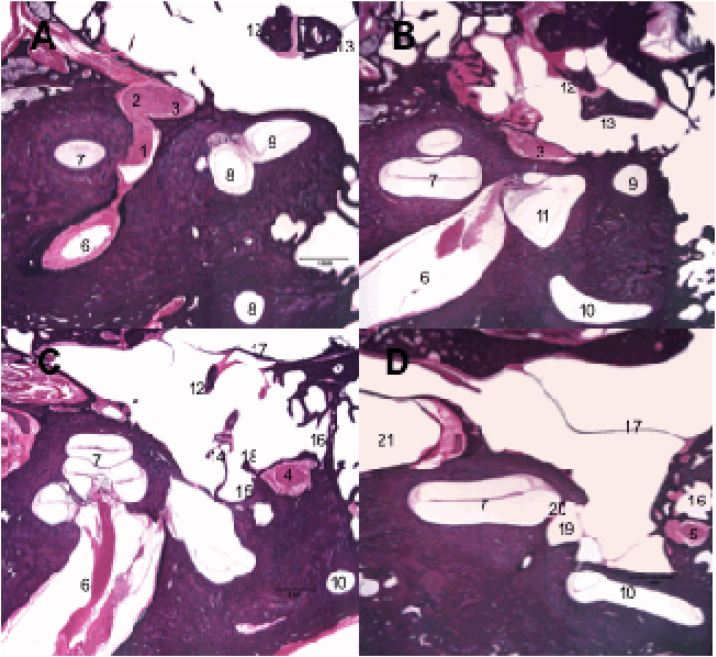
Histological sections of temporal bone at 20× magnification, right ear, stained with hematoxylin & Eosin. 1: Facial nerve, labyrinthine segment; 2: Geniculate ganglion; 3: Facial nerve, tympanic segment; 4: Mastoid segment of the facial nerve; 5: Facial nerve, mastoid segment; 6: Internal auditory canal; 7: Cochlea; 8: Superior semicircular canal; 9: Lateral semicircular canal; 10: Posterior semicircular canal; 11: Utricle; 12: Malleus; 13: Incus; 14: Stapes; 15: Tympanic sinus; 16: Facial recess; 17: Tympanic membrane; 18: Pyramidal eminence; 19: Oval window niche; 20: Oval window; 21: Carotid artery.

Along its intratemporal segment, the facial nerve is in close proximity to several structures of the inner and middle ear (Fig. 3). The labyrinthine segment begins at the end of the IAC and is the shortest of the intratemporal segments, with an average of 3–5 mm in length, and is also the narrowest segment.^9^, ^16^ It extends from between the cochlea and the posterior labyrinth to the region of the geniculate ganglion, where it curves to create an angle of approximately 70 degrees, referred to as the first genu of the facial nerve.^17^, ^18^ At this point, the greater superficial petrosal nerve emerges and innervates the lacrimal glands, being responsible for lacrimation.

The tympanic or horizontal segment is the second segment of the facial nerve, with an average of 7 mm in length, and is located between the first and second genu. It is the most exposed segment of the facial nerve, with several areas of dehiscence of the bony canal, making it more susceptible to injury. From the first genu, at the level of the cochleariform process, the nerve passes through the middle ear cleft, running very close to the oval window, until it curves to create an angle of approximately 90 degrees medial to the lateral semicircular canal, considered the second genu of the facial nerve.^18^

The mastoid or vertical segment is the longest intratemporal segment, running from the second genu to the exit of the stylomastoid foramen, and is usually over 12 mm in length. This segment gives off two branches: the nerve to the stapedius muscle and the chorda tympani nerve. The nerve to the stapes, responsible for the stapedial reflex, usually emerges from the mastoid segment near the second genu. However, in some individuals, it may originate from the distal tympanic segment.^15^, ^19^ The chorda tympani nerve arises from the most distal mastoid segment and crosses the tympanic cavity to join the lingual nerve, providing taste function to the anterior two-thirds of the tongue, in addition to carrying parasympathetic fibers to the submandibular and sublingual glands.^15^

The function of the three intratemporal branches of the facial nerve allows the clinical topographic diagnosis of nerve injury by evaluating lacrimation (dry eye sensation – Schirmer test), stapedial reflex (hyperacusis – immittance testing), and taste (tested with salt and sugar on the anterior two-thirds of the tongue on the affected side).

Intratemporal nerve injury, regardless of the etiology, results in edema and subsequent increase in endoneurial fluid pressure.^20^ It is believed that this increased pressure within the perineurium, which is poorly compliant, constricts the epineural and transperineural vessels, with impairment of blood flow within the *vasa nervorum* and subsequent nerve injury due to ischemia.^21^, ^22^ It is assumed that in facial neuritis, of traumatic or infectious etiology, the phenomena that occur within the facial nerve canal may lead to compression of the facial nerve and compromise of the blood supply, causing ischemia and axonal degeneration. Bell’s palsy, for example, is still the most common facial palsy and generates much discussion about the factors that may predispose to this condition, including anatomic differences. Studies using imaging techniques and temporal bone compared the diameter of the bony facial canal between patients with Bell’s palsy and controls and found statistically significant differences, suggesting that there may be a relationship between narrower canals and Bell’s palsy.^17^, ^23^, ^24^, ^25^ In addition to the importance of such studies to the etiology of PFP, they also have implications for surgical treatment by indicating more precisely the possibility of facial nerve decompression and the segments to be decompressed.

The extratemporal segment begins as the facial nerve exits the stylomastoid foramen. After exiting the stylomastoid foramen, the facial nerve gives off two branches: the posterior auricular nerve and the digastric nerve (supplying the posterior belly of the digastric muscle). Just before entering the parotid gland, the main trunk of the facial nerve divides into the superior (temporofacial) and inferior (cervicofacial) trunks, which give rise to the main five branches that innervate the facial muscles: temporal, zygomatic, buccal, marginal mandibular, and cervical branches (Fig. 2). From exiting the stylomastoid foramen to reaching the facial muscles, the facial nerve is 6–9 cm in length. Along its course, it is related, in addition to the parotid gland, to the parotid duct, submandibular gland, mandibular branch and condyle, and Bichat ball.^18^

In extratemporal facial palsy, it is important to consider the relationship of the facial nerve with these structures in the diagnostic hypothesis of a tumor that may affect the facial nerve due to dissemination of neurotropic tumor cells or nerve compression. Iatrogenic facial nerve palsy should also be considered in cases of dental procedures with anesthesia and orthognathic surgery.

After passing between the superficial and deep lobes of the parotid gland, the facial nerve branches vary in depth in each segment, being deeper in the cervical region, below the platysma, and more superficial in the face, located just below the Superficial Musculo-Aponeurotic System (SMAS). These differences should be taken into account in surgical and cosmetic procedures of the face to avoid transient or permanent iatrogenic injury.

### Pathophysiology

Fig. 1 illustrates the anatomy and histology of a transected facial nerve surrounded by three supporting structures known as endoneurium, perineurium and epineurium, thus forming the nerve trunk.^26^, ^27^

After injury (of any nature) to a peripheral nerve, a sequence of cellular events takes place, dependent on the severity of the injury and the proximity of the injured segment to the cell body.^28^, ^29^ This process aims at the degeneration of injured axonal segments, mediated by the recruitment of macrophages, and regeneration of the axon resulting from the activation of Schwann cells.^28^

This process is called Wallerian degeneration and occurs within 24 h post-injury. The primary change in Wallerian degeneration is the fragmentation of the axon in the proximal segment, connected to the cell body, and in the distal segment. In this process, both the neurotubules and neurofilaments from the cytoskeleton framework of both stumps become disarrayed and retract. At the same time, the nucleus (which is in the cell body connected to the proximal stump of the nerve) migrates to the periphery of the cell, and Nissl bodies (or granules) (clusters of rough endoplasmic reticulum) break up and disperse, a process known as chromatolysis.^26^, ^27^, ^28^, ^29^

This event lasts 10–21 days and, as previously mentioned, is triggered by injury (of any nature) to the nerve, being the precursor of the regeneration cascade or apoptosis. In cases of axonal injury (when the cell body and nucleus are spared), peripheral chromatolysis occurs, where Nissl bodies are initially lost at the periphery of the nerve with subsequent progression to the nucleus.^30^ The debris resulting from this disintegration is then phagocytosed by macrophages aided by Schwann cells.^31^, ^32^, ^33^, ^34^ These cells proliferate rapidly within the first 24 h post-injury and trigger the upregulation of protein genes that assist in cell degeneration and regeneration.^31^, ^32^, ^34^

By 48–96 hours post-injury, axonal structures and local myelin are disintegrated by Wallerian degeneration. Nerve conduction is completely lost, and in severe injury, regeneration begins only after Wallerian degeneration has run along the entire length of the injured nerve.^28^ The first signs of the regeneration process are visible within 24 h post-injury.^28^ Changes in the cell body mark the reversal of chromatolysis, reprogramming the metabolic processes to produce proteins and lipids needed for axonal regeneration. Simultaneously, proliferated Schwann cells (which also help macrophages to remove cell debris resulting from chromatolysis) provide the formation of the cytoskeletal structures that connect the two stumps of the transected nerve.^9^, ^26^, ^28^ During this stage, some axonal sprouts may be misdirected, sprouting into endoneurial tubes other than their own. If multiple axons are misdirected, erroneous reinnervation occurs and other areas not related to the course of the injured nerve begin to be stimulated. Clinical examples of this process are mass movement and synkinesis.^26^, ^28^

The success of regeneration depends on the degree of nerve injury. In 1943, Seddon described three basic types of lesions: neurapraxia, axonotmesis, and neurotmesis.^35^ In 1950, Sunderland histologically expanded this classification to five degrees (I–V) of nerve injury (Table 6).^34^, ^36^

**Table 6:** Classification of facial nerve lesions and histological characteristics according to Sunderland.

Seddon	Sunderland	Characteristics
Normal	Normal	No changes
Neurapraxia	Grade 1	Axonal conduction block without damage to the neural structure There is complete recovery, without sequelae
Axonotmesis	Grade 2	Axonal injury leading to Wallerian degeneration with breakdown of the myelin sheath, but the nerve structure is not damaged. Full recovery is typically achieved.
Neurotmesis	Grade 3	Axonal and endoneurial injury: in addition to Wallerian degeneration, there is disruption within the endoneurium with the possibility of fibrosis, which leads to a worse recovery prognosis in relation to grades II and III
	Grade 4	Axonal + endoneurium + perineurium injury: with only the epineurium intact, there is great neural disruption, with loss of fascicle continuity. In addition to fibrosis that prevents the axon stumps from meeting, this also allows axonal growth toward neighboring fascicles, determining the appearance of synkinesis + Grade 4 lesions cause important sequelae, including synkinesis.
	Grade 5	Axonal + endoneurium + perineurium + epineurium injury: there is loss of nerve trunk continuity. In this case, there is no spontaneous recovery.

Degree I (the mildest degree of this classification) is essentially the same as neurapraxia of Seddon’s classification. This degree corresponds to a temporary nerve conduction block, not being related to any degree of axonal injury (with complete recovery and without sequelae).

Degree II corresponds to axonotmesis. It is important to highlight that, in this degree of nerve injury, by both classifications, axonal injury is accompanied by degeneration of the myelin sheath, but with preservation of the endoneurium and supporting structures of the nerve (generally, recovery is also complete).

Degrees III and IV were added by Sunderland and are not described in Seddon’s original classification. Degree III is characterized by axonal and endoneurial injury, with the possibility of fibrosis development, but with preservation of the perineurium. Degree IV corresponds to damage to the axon, endoneurium, and perineurium, but with preservation of the epineurium. There is extensive nerve disruption, with disorganization of the nerve fascicles, leading not only to the development of fibrosis (which prevents the axon stumps from meeting again) but also to disorganized axonal growth, allowing the occurrence of synkinesis.

**Figure 4:**
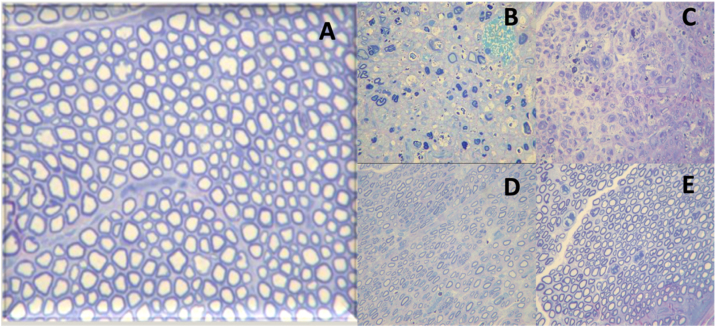
Histological images of the evolution of an axonotmesis-like lesion of the facial nerve. Axonotmesis: facial nerve injury by compression and evolution at 6 weeks. (A) Normal facial nerve. (B) Facial nerve 1 week after the injury showing significant degeneration, with loss of the myelin sheath. (C) Facial nerve 2 weeks after injury. (D) Facial nerve 4 weeks after injury. (E) Facial nerve 6 weeks after the injury with a structure already quite similar to the normal facial nerve.

Finally, degree V corresponds to neurotmesis. In this degree, there is nerve transection with complete loss of histological structures (axon, endoneurium, perineurium, and epineurium). A representation of axonotmesis is shown in Fig. 4.

## Objective

This systematic review aims to make evidence-based recommendations for the diagnosis and treatment of PFP.

## Methods

In August 2022, a task force consisting of otolaryngologists, otology specialists, Brazilian Society of Otology (Sociedade Brasileira de Otologia, SBO) directors, and SBO members met in person and remotely to discuss the topic of this guideline. Each participant in this meeting was tasked with giving a 15- to 30-minute evidence-based lecture on one of the suggested topics. After the lecture, the participants discussed the topic until reaching a consensus. Each author was asked to write a text with the current literature on the topic, based on evidence and containing the elements discussed during the meeting. A rapporteur prepared the final text, which was reviewed by the other coauthors and the Brazilian Journal of Otorhinolaryngology (BJORL) editor.

The recommendation methods of this guideline follow those of the American College of Physicians (ACP) and the American Thyroid Association (ATA), as previously published by the authors of this task force.^37^, ^38^, ^39^, ^40^, ^41^

This guideline is not intended to be a substitute for individual professional judgment. Physicians should always act and decide in a way that believe is best for their patients, regardless of guideline recommendations. They should also operate within their scope of practice and in accordance with their training. The guidelines represent the best judgment of a team of experienced physicians addressing the scientific evidence for a given topic.

## Evidence-based evaluation and diagnosis of PFP

### Electrophysiologic tests in the diagnosis of PFP

Interest in electrophysiologic studies of the facial nerve was introduced by Esslem and popularized by Fisch in the 1970s.^42^ Since then, advances have included their use in the diagnosis and treatment of PFP, particularly because they may add to the prognosis of a disease that has important anatomic and functional impact on affected individuals.^1^, ^42^, ^43^, ^44^

Neural signal conduction through the axons toward the muscle cells occurs because of the generation of an electrochemical current that reflects changes in sodium and potassium concentrations in the intracellular and extracellular spaces. These electrochemical changes from a resting state (resting potential) to an active state (action potential) can be studied in several ways. The knowledge produced has been used in clinical practice in patients with PFP to investigate neural signal transmission to the muscle cells. Known as electrodiagnostic testing of the facial nerve, the tests can be used to check not only the conduction state of the nerve but also the course of facial palsy.^45^

Chronologically, several tests have been well established in the literature and applied clinically. Some will be reported here only because of their historical value, although they are no longer used, since they have been replaced by more accurate assessments.

### Conduction velocity and latency testing (LAT)

In 1965, Taverner^46^ described one of the first methods for electrodiagnosis in facial palsy. According to the author, the nerve should be stimulated at the stylomastoid foramen and the time for muscle contraction should be measured using an oscilloscope. A threshold of 4 ms is considered the upper limit of normal. In cases of peripheral nerve involvement, this latency increases. Within seven days of the onset of the facial palsy, it indicates denervation and development of sequelae. To calculate conduction velocity, the parameter is the length of the nerve.

Over the years, it became clear that this technique was best suited for long nerves, such as limb nerves, with limited application to the facial nerve whose extratemporal course is short. Therefore, the test was abandoned after an unreliable correlation was observed in several cases of peripheral nerve injury.^47^

### Electromyography (EMG)

In 1944, EMG began to be used to evaluate the amplitude of the contraction of the facial muscles with an electrical stimulus or voluntary muscle contraction. Two techniques are used – needle EMG (with needle electrodes to study groups of muscle fibers) and surface EMG (with surface electrodes to study muscle groups).

In practice, in needle EMG, possible responses are as follows: (a) Silence or rest, which can occur in innervated muscles normally at rest or with intense fibrosis and/or degeneration; (b) Motor unit action potential, normally present during voluntary muscle contraction. This action potential is absent in facial palsy; (c) Fibrillation potential present in muscle fibers with ongoing denervation; (d) Polyphasic potentials present during nerve regeneration in which four or more phases of muscle potential appear.

In the acute stage of facial palsy, 10–21 days post-injury, invisible muscle fiber contractions (fibrillations) occur. Polyphasic potentials occur after this phase and at the beginning of regeneration. This test has an important application in the clinical follow-up of facial palsy and decision-making, but some limitations need to be pointed out.^45^, ^47^

When performed with needle electrodes, only a few groups of fibers are analyzed, which may lead to misinterpretation of good or poor prognosis, depending on the random selection of the site for needle insertion. The test provides a longitudinal evaluation over time, requiring at least two tests to be performed during the facial palsy. According to data from previous analyses, the effects of nerve injury on the myoneural plaque appear after 10–14 days, which certainly confuses the results in cases of early testing, leading to false expectations in the setting of a poor prognosis.

Surface EMG is an easy-to-perform test that can be used in offices or as biofeedback for speech training.

### Minimal nerve excitability testing (NET)

Electrical currents are applied to the nerve trunk, at the stylomastoid foramen, and the intensity is progressively increased until a visible facial muscle contraction can be detected or the maximum intensity has been reached, neither of which occurs if the facial nerve is injured. The main purpose of the test is to detect nerve degeneration early. Improvements over time have allowed testing of the main five branches of the facial nerve. A difference greater than 3.5 ms between the affected side and the normal side is indicative of degeneration. The test gained popularity after a stimulator with an excellent cost-benefit ratio was presented by Hilger in 1964.^48^

In 1972, Mark May reported several limitations of the test based on a study of 130 patients with PFP of various etiologies.^49^ Given the phenomenon of neural desynchronization that occurs during nerve regeneration, patients with no responses had a good final prognosis.

### Maximal stimulation test (MST)

Aiming to improve prognostic accuracy, May et al.^50^ proposed, in 1971, a change in the protocol of electrical stimulation testing to use maximal rather than minimal stimulation, which would lead to increased intensity of facial muscle response to electrical stimulation. When comparing the responses on the affected side vs the normal side, possible outcomes are as follows: (a) Normal for equal responses; (b) Slightly decreased; (c) Greatly decreased; (d) No response.

The authors reported the results for 42 patients with Bell’s palsy evaluated on days 3, 7, 10, and 14 after the onset of the facial palsy. When the test was normal by day 10, 92% of patients recovered completely. When altered, 100% of patients had significant sequelae. In practice, although some physicians still use the Hilger stimulator in their offices, this evaluation relies on a subjective qualification of the muscle response after electrical stimulation.^48^

### Electroneurography (ENoG)

This test emerged in the 1970s, initially described by Esslen and later popularized by Professor Fisch’s publications as a result of the need to improve prognostic accuracy in patients with facial palsy.^42^, ^43^, ^44^, ^51^ Since then, the test has had several names, but the use of the term Electroneurography (ENoG) is recommended in daily clinical practice.

ENoG relies on an evoked, supramaximal electrical stimulus delivered at the stylomastoid foramen to activate the ipsilateral facial nerve.^52^ Electrical stimulation of the facial nerve performed distal to the site of injury generates a Compound Muscle Action Potential (CMAP), which is the summation of the various action potentials of a muscle group. The CMAP amplitude is dependent on the synchronous discharge of viable nerve fibers. Reduction in CMAP amplitude is associated with Wallerian degeneration of the nerve.^52^ The CMAP amplitude on the affected side is compared with the CMAP on the normal side, which serves as a control, and a percentage of degenerated nerve fibers is calculated.^52^ This potential is reflected in the number of viable axons that reach this muscle group. When the facial nerve is stimulated distal to the site of injury, if there is transmission of nerve impulses by the axons, regeneration is possible, being called neurapraxia. This conduction is absent in axonotmesis and neurotmesis.

To perform ENoG, it is crucial to wait for the period of nerve degeneration that occurs within 10–14 days of the onset of the facial palsy. This test has a comparative nature and investigates, through CMAP amplitudes, the number of viable axons that reach the muscle group under study.

The test proportionally evaluates the number of normal axons on the normal side compared with the side with facial palsy and expresses the number of viable axons as a percentage. ENoG is one of the most recommended tests for the follow-up of patients with PFP of any etiology.

ENoG differs from EMG in that EMG evaluates individual muscle unit potentials, while ENoG is a summation muscle action potential. Normal reference values vary greatly for potential latency, amplitude, and test/retest fluctuations.

The validation of the test was reported in 1980 by Adour,^53^ who described the following normal values for ENoG: nerve conduction latency (2.48 ± 0.38 ms), CMAP amplitude (2.38 ± 0.73 mV), and test/retest fluctuation (16%) (Table 7).

**Table 7:** Reference values for electroneurography.

Variable	Upper limit of normal	SD
Nerve conduction latency	2.48 ms	±0.38
CMAP amplitude	2.38 mV	±0.73
Test/retest fluctuation	16%	–

CMAP, Compound Muscle Action Potential; ms, Milliseconds; Mv, millivolts.

In daily clinical practice, the following precautions are recommended in the interpretation of ENoG:

1) Obesity is associated with a reduction in CMAP amplitude due to equipment limitations, leading to a greater need for a comparison between the two sides of the face;
2) In situations of intense stimulation, other muscle groups may compose the facial CMAP response, thereby resulting in a triphasic response produced by a contraction of the masseter and pterygoid muscles. Therefore, this should be considered when interpreting the results, as there is not always a description of the test methodology, and the attending physician needs to be aware of that;
3) Tracing with few artifacts is desirable. The use of an electrical network with a proper grounding system is recommended;
4) During the period of nerve regeneration, neural desynchronization also occurs. Thus, muscle depolarizations occur at different time points, and it is not always possible to detect the ENoG summation potential. Follow-up with clinical examination associated with electrodiagnosis is essential;
5) ENoG should be performed comparatively for the prognostic evaluation of facial palsy. Good interaction is required between those performing the test and those interpreting it and caring for patients with facial palsy;
6) Some situations may limit or even discourage the use of the test: complete transection of the facial nerve (the process of nerve degeneration is a definite outcome of the already established injury); and recurrent facial palsy (since the test requires a comparison between the two sides and is highly dependent on the technique employed). In the latter situation, the prognostic value of the test becomes compromised, especially in cases where recurrence occurs on the previously healthy side;
7) The test may be indicated in patients with Bell’s palsy. However, as most of these patients progress well with medical treatment, the test is recommended once the paralysis has become complete and in cases where the condition lasts beyond three weeks.

Although the otolaryngologist often does not perform the test, the reports usually do not provide values of current intensity or setting parameters. Comparative repetition of tests will only be relevant for follow-up if performed by the same team. Preparation of the patient’s skin, placement of the electrodes and stimulation probe, and stimulation intensity all contribute to a good performance of the test.

### Electroneuromyography after temporal bone fracture

The most widely used complementary assessments in clinical practice that provide the greatest amount of information about the degree of nerve injury are Nerve Conduction Studies (NCSs) and EMG. In many centers, both are performed simultaneously and, therefore, called electroneuromyography.^54^

NCS (performed from electrical nerve stimulation) is a rapid and efficient method to quantify the latency of potentials and, consequently, the Nerve Conduction Velocity (NCV) and the amplitude of both sensory and motor action potentials of the nerve. Latency and NCV reflect speed of propagation of action potentials by saltatory conduction of impulses along the myelin sheath. The amplitude of the motor action potential reflects integrated function of the motor axons, motor end plate, and striated muscle.^54^ Thus, it is possible to differentiate myelination events, in which there is predominantly a prolonged latency and reduced velocity, from axonal events, in which, for example, there is a reduction in the amplitude of potentials.

Needle EMG (performed with needle electrodes in a specific muscle group) allows us to differentiate muscular (myopathic) from neurogenic changes, in addition to providing temporal data (differentiation between acute, subacute, and chronic patterns) using, to this end, the activation pattern of the assessed muscle and the presence or absence of ongoing denervation activity.^55^, ^56^ Although it is a pathological classification, the degree of injury can be inferred from neurophysiological data (Table 8).^55^, ^56^

**Table 8:** Summary of neurophysiological findings in neural lesions.

		Distal latency	Conduction velocity	CMAP amplitude	EMG
Myelin		Increased	Decreased	Normal or slightly decreased (except in conduction block)	Normal
Axonal	Axonotmesis	Normal or slightly altered	Normal or slightly altered	Decreased	Denervation and remodeling of the remaining units
	Neurotmesis	Absent	Absent	Absent	Presence of denervation, no voluntary activation

CMAP, Compound Muscle Action Potential; EMG, Electromyography.

Patients with neurapraxia tend to have only myelin damage with prolonged latency and reduced velocity, without significant changes on needle EMG. Patients with axonotmesis tend to have an associated decrease in amplitude and varying degrees of changes on needle EMG, with signs of acute denervation activity and remodeling of the remaining units. In neurotmesis, the nerve is not excitable (lack of potential), with extensive denervation activity and absence of voluntary activation on needle EMG.^55^, ^56^

A peculiar situation occurs in conduction block in myelinated axons: cases in which severe neurapraxia temporarily leads to blockade of nerve transmission with a reduction in the amplitude of potentials. It differs from neurotmesis in that there are no changes on needle EMG examination, indicating no damage to the axon, and there is recovery of the amplitude of potentials in subsequent examinations. The combination of these data allows locating the lesion and defining its substrate (axonal or myelin damage), severity, and temporality (acute, subacute, or chronic).^55^, ^56^

However, it is relevant to note that changes in the electroneuromyography examination depend on the Wallerian degeneration process, that is, the distal nerve degeneration that occurs after an axonal injury. Wallerian degeneration occurs typically between 3 and 5 days (for motor fibers) and between 6 and 10 days (for sensory fibers). Therefore, only 10–14 days after the event, the degree of axonal loss can begin to be adequately assessed.^55^, ^56^

Nerve remodeling initiates at the very beginning of the Wallerian degeneration process, and some fibers may have completed the entire process within three weeks of injury. After an axonal injury, the first signs to appear on EMG recordings are typically of brief duration (decreased activation), polyphasic, small, and of a low amplitude. As the nerve regenerates, they become more abundant until the pattern of a completely reintegrated nerve is present, with potentials of high amplitude, long duration, and polyphasic, but with a decreased recruitment pattern, since there was an effective reduction in the number of motor units.^26^, ^55^, ^56^

Although the pathophysiology of the disease suggests that examinations should ideally be performed three weeks after the injury (after Wallerian degeneration), examinations performed in the first week can be of great value. During this period, detection of the axon discontinuity conduction block can precisely identify the site of injury. This information is important in cases of extensive trauma. Another advantage of an early study is to determine whether the lesion is electrophysiologically complete or incomplete, which determines prognosis and the likelihood of surgical intervention.^26^

After one or two weeks, electrodiagnosis can determine whether the deficit is due to neurapraxia or a more severe axonal injury. Fibrillations appear after three to four weeks. Studies performed at three to four months after the injury may detect signs of reinnervation.^26^

Finally, in cases of axonotmesis, it should be noted that the so-called nerve viability index can be provided or calculated. It is the ratio of the amplitude of the potential on the affected side to the normal side. Rates close to 1 (or 100%) indicate a mild lesion, while rates close to 0 indicate an almost complete lesion, which may correlate with a worse prognosis.^55^, ^56^

Most of the clinical observations and validations derive from tests performed in patients with Bell’s palsy, so studies in populations of patients with other etiologies of facial palsy are extremely important.

There is no infallible test for the prognostic evaluation of PFP cases. The study of CMAP latency and speed of propagation is considered to have little clinical application in view of the short extratemporal course of the facial nerve, so it is not recommended. Minimal NET, although still widely used by otolaryngologists in their offices, has a dubious correlation with the prognosis of facial palsy.^49^ It may be recommended based on the difficulty in performing more accurate tests. MST is the best option for assessing prognosis, but due to the subjective interpretation of responses, its application has been criticized in daily practice.^57^ It may be recommended based on the difficulty in performing more accurate tests or unavailability of more objective tests. ENoG, after its validation as an electrodiagnostic method, is considered the best and most objective prognostic test for patients with facial nerve dysfunction, being recommended for the prognostic evaluation of PFP.^58^

**Figure 5:**
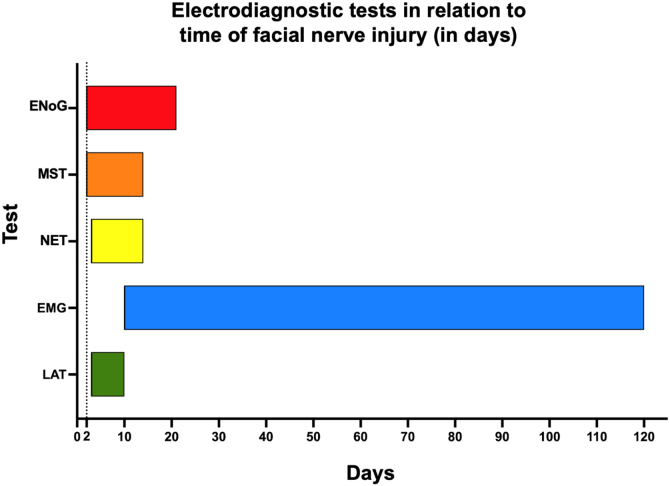
Indication of electrodiagnostic tests according to the degree of evolution of peripheral facial palsy. EMG, Electromyography; ENoG, Electroneurography; LAT, Latency test; MST, Maximal Stimulation Test; NET, Nerve Excitability Test.

Needle EMG allows the assessment of the onset of nerve recovery as well as degeneration. For an objective functional assessment, surface EMG is well indicated. Regarding the temporality of PFP progression, Fig. 5 shows the potential applications of electrodiagnostic testing. This method is recommended for the follow-up of PFP cases during recovery.

### Recommendations

I – ENoG is considered the best and most objective prognostic test for patients with PFP, being recommended for the prognostic evaluation of PFP (Strong recommendation; Moderate-quality evidence).

II – Within 7–14 days of the onset of facial palsy, any type of electrodiagnostic testing for more definitive conclusions should be avoided (Strong recommendation; Low-quality evidence).

III – In the acute phase, response latency detection, minimal NET, MST, and ENoG may be indicated (Moderate recommendation; High-quality evidence).

IV – In patients with traumatic facial palsy, during the course of otitis media, or resulting from intraoperative iatrogenic injury, electrophysiologic tests play a crucial role due to the importance of establishing the need for a surgical approach and/or re-approach, as well as the optimal timing for that procedure (Strong recommendation; High-quality evidence).

V – Despite the recommendation of the American Academy of Otolaryngology-Head and Neck Surgery (AAO-HNS) guidelines in cases of Bell’s palsy, electrophysiologic tests may be optionally performed, according to the demand of patients or physicians for an objective prognostic evaluation. They are recommended for the follow-up of patients with no signs of progression after three weeks post-injury (Strong recommendation; Low-quality evidence).

VI – In cases of recurrent facial palsy, the test may have low sensitivity and low accuracy, and is occasionally recommended (Insufficient evidence).

## Idiopathic PFP (bell’s palsy)

Bell’s palsy is an acute PFP, usually partial, but it may present as a complete palsy, of unknown cause. It is important to emphasize at this point that this is a diagnosis of exclusion.

It results from inflammation and dysfunction of the facial nerve. While the exact etiology of Bell’s palsy remains unknown, there is evidence that it may be related to viral infections. Some viruses have been implicated in Bell’s palsy, such as HSV, VZV, Epstein–Barr Virus (EBV), Cytomegalovirus (CMV), Respiratory Syncytial Virus (RSV), and influenza viruses.^59^ Risk factors associated with the development of Bell’s palsy include pregnancy, obesity, and diabetes, among others.^60^ Bell’s palsy can affect any age group, but it is more common between the ages 15 and 45 years. Whereas early pregnancy is associated with a decreased incidence of Bell’s palsy, the third trimester of pregnancy and the immediate puerperium are associated with a 2- to 4-fold increase.^60^, ^61^, ^62^, ^63^, ^64^, ^65^, ^66^ Compared with cases unrelated to pregnancy, Bell’s palsy in pregnancy is associated with worse long-term outcomes to a degree that cannot be explained by differences in medical therapy alone.^67^

Bell’s palsy often has a good prognosis. Most patients show improvement within two to three weeks and complete recovery by three to four months. Approximately 30% do not fully recover.^59^ Among pregnant women, complete recovery of facial function also occurs in approximately 70% of cases.^68^

### Etiology/pathophysiology

By definition, the cause of Bell’s palsy is uncertain. However, it is believed that reactivated herpes virus in the geniculate ganglion region may play a key role in the development of Bell’s palsy.

Some studies have demonstrated the presence of herpes virus in patients with Bell’s palsy. Herpes Zoster (HZ)-associated facial palsy more frequently presents as Zoster Sine Herpete (ZSH), without vesicles, although 6% of people develop vesicles (Ramsay Hunt syndrome).^69^ Furuta et al.^70^ evaluated 121 patients clinically diagnosed with Bell’s palsy by Polymerase Chain Reaction (PCR) analysis and detected viral reactivation without the presence of vesicles in 29% of patients. Kawaguchi et al.^69^ investigated the presence of HSV and VZV and found viral reactivation in 34% of patients diagnosed with Bell’s palsy. Therefore, management of Bell’s palsy should take into account the possibility of VZV infection, even when there is no typical presentation of the virus.

As in adults, the most common etiology in children is idiopathic, accounting for approximately 65.4% of cases.^71^ Potential infectious etiologic agents include adenovirus, VZV, EBV, *Mycobacterium tuberculosis*, *Borrelia burgdorferi*, *Haemophilus influenzae*, CMV, rubella, mumps, *Mycoplasma pneumoniae*, and HIV, as well as complications of acute otitis media and cholesteatomatous chronic otitis media.^3^, ^72^

In cases of pregnant women, there is evidence that facial palsy in late-term pregnancy and the immediate puerperium is a risk factor for worse long-term facial function outcomes.^67^ In addition, the rate of gestational hypertension and pre-eclampsia is higher in pregnant women with Bell’s palsy than in the general obstetric population.^73^, ^74^ Some physiological changes occurring during the third trimester of pregnancy have been considered possible reasons for this increased incidence, including relative immunosuppression by elevated cortisol levels, increased susceptibility to viral infections and HSV reactivation, increased extracellular volume, and prothrombotic states.^60^, ^63^, ^66^, ^75^

### Clinical presentation

Although Bell’s palsy usually affects only one side of the face, in rare cases it can affect both sides. Bell’s palsy symptoms usually develop rapidly within two to three days.

As the facial nerve is a mixed nerve, with motor and sensory branches, the main clinical manifestation of nerve involvement is the impossibility of voluntarily moving the facial muscles on the side ipsilateral to the injured nerve. To a lesser extent, other signs and symptoms may occur, such as mild pain in or behind the ear, oropharyngeal or facial numbness, impaired tolerance to ordinary levels of noise, and disturbed taste on the anterior part of the tongue. Severe pain is more suggestive of HZ infection (Ramsay Hunt syndrome). More recently, an association of facial palsy with SARS-CoV-2 infection (COVID-19) has been reported, with a higher incidence of facial palsy in patients who have been infected with SARS-CoV-2. The underlying mechanism of facial palsy after COVID-19 is likely to be molecular mimicry attributable to a neuroimmunological process between microbial and nerve antigens.^76^ Facial palsy in these patients may occur as part of a broader syndrome, such as Guillain–Barré syndrome, or alone.^77^

It is important to note that a major repercussion related to facial palsy is the inability to close the eyelids, leading to potential eye damage.

Although most cases of Bell’s palsy resolve completely, some patients may have residual complications such as involuntary movements or spasms of the facial muscles on the affected side and the shedding of tears while eating or drinking (crocodile tears) due to abnormal nerve recovery.

### Diagnosis

A detailed history with information about potential triggering factors, associated symptoms and history of viral infection is important to help identify possible etiologies and exclude other causes of facial palsy. In addition, it helps identify the comorbidities that may be related to prognosis and factors that may interfere with the indication of medical treatment.

Physical examination is extremely important to initially make the differential diagnosis between PFP and central facial palsy. In PFP, facial muscle function, at rest and movement, is affected throughout the side ipsilateral to the injured nerve. In central facial palsy, facial muscle function is usually preserved in the upper third of the face on the affected side (except in cases of central etiology after the facial nucleus, which can affect all facial muscles). In addition to the specific assessment of facial palsy, the physical examination should include a complete ear, nose, and throat evaluation. Careful inspection of the external auditory canal, tympanic membrane, parotid gland, and skin of the head and face is essential in the search for abnormalities that may lead to the etiologic diagnosis. Physical examination of the parotid gland region is crucial, as lesions spreading to the main trunk of the facial nerve after its intraparotid portion may cause facial palsy while preserving the upper third of the face, mimicking a paralysis of central origin. Atypical signs and symptoms of Bell’s palsy, such as bilateral involvement of the facial nerve, complete paralysis, and recurrence, warrant an active and comprehensive investigation.

Initially, laboratory testing is not required.^78^ This is due to the low detection rates of HSV or VZV, even with the use of PCR, Enzyme-Linked Immunosorbent Assay (ELISA), Western blot, and Cerebrospinal Fluid (CSF) analysis.

### Laboratory testing

Laboratory tests should be ordered if the history-taking or physical examination reveals any data that indicate a specific etiologic factor.

History of any recent viral infection should be investigated (COVID-19, herpes, and mononucleosis, among others), as well as any suspected abnormality in the physical examination or symptoms that may guide an investigation by serologic testing or PCR. For patients in endemic areas (or patients who have recently traveled to endemic areas), samples should be collected for serologic testing for Lyme disease, particularly when the patient’s history is suggestive of exposure.

### Imaging studies

The routine use of diagnostic imaging is not recommended at the time of initial presentation of these patients. Although Magnetic Resonance Imaging (MRI) studies of Bell’s palsy may commonly show enhancement along the involved (ipsilateral) facial nerve – especially around the geniculate ganglion region – this finding does not influence the course of therapy. However, if there are any signs of an atypical course of the disease, such as failure to recover within the expected time frame, bilateral or recurrent paralysis, or paralysis of isolated branches, involvement of other cranial nerves, or any other features atypical of Bell’s palsy, imaging of the entire course of the facial nerve is recommended.

The imaging modality of choice is contrast-enhanced MRI, with Computed Ttomography (CT) being reserved for cases where MRI is not possible or with a history of trauma.^78^ There is no recommendation for the use of imaging in Bell’s palsy, which essentially remains a clinically diagnosed disease. If there is concern about differential diagnoses when other etiologies are suspected, MRI and CT can be used (Table 9). There are natural concerns with the use of diagnostic imaging during pregnancy. However, when needed during pregnancy, the imaging technique of choice is MRI due to the ability to image deep soft tissue structures without posing a risk to the fetus or the pregnancy.^79^ The use of gadolinium remains controversial and should be limited to cases in which its use considerably improves diagnostic performance. Traditionally, there has been fear of radiation-induced teratogenesis. Head or neck CT has been associated with “very low-dose examinations (<0.1 mGy)” and should not be withheld from a pregnant patient if MRI is not readily available or additional imaging is required.^79^

**Table 9:** Indications for imaging in acute facial palsy.

1. Complications of chronic ear diseases (i.e., cholesteatoma)
2. Palsy that progresses after 3 weeks
3. Recurrent facial palsy
4. Any patient contemplating surgical intervention
5. No functional improvement after 3 months
6. Asymmetric weakness across facial zones
7. Additional cranial neuropathies or focal neurologic deficit

### Electrodiagnostic testing

In patients with a typical clinical picture of Bell’s palsy with incomplete facial palsy, it is not recommended to routinely order electrodiagnostic testing.^78^

Electrodiagnostic testing should not be used in the management of Bell’s palsy because, for most patients, the chances of complete recovery are very high and electrodiagnostic tests provide no direct benefit to treatment or diagnosis, in addition to the high cost and discomfort of the procedure. However, in cases of complete facial palsy or poor recovery, electrodiagnostic testing may provide prognostic information and help identify potential surgical candidates.^80^

Electrodiagnostic testing poses no harm to the patient who is pregnant or breastfeeding. Tests can be performed four days after the onset of facial palsy. There is a delay in detecting nerve degeneration through this test considering the onset of nerve injury. This occurs because inferences are made based on the nerve distal to the site of injury. Excellent recovery of facial function occurs when the decline in the CMAP, as measured by ENoG, does not reach 90%. Half of the patients who reach this level of degeneration also have excellent outcomes.^78^ Responses less than 10% of those of the contralateral side predict a poorer response regardless of treatment, which is helpful for prognosis and early referral to facial physical therapy and a facial nerve specialist. Based on these findings, it is recommended that only patients with complete palsy undergo electrophysiologic testing.^78^

### Treatment

Treatment of PFP aims at complete or partial recovery of facial nerve function, especially of motor activity with the recovery of facial muscle function. It also aims to prevent the progression of facial palsy from partial to complete, reduce the incidence of motor synkinesis and contracture, and reduce the risk of eye damage.

Although there is a strong tendency for medical treatment to be withheld in pregnancy-associated cases of Bell’s palsy, factors intrinsic to pregnancy appear principally responsible for the worse prognosis. Avoiding the use of systemic corticosteroids in the first trimester of pregnancy is prudent. However, use of corticosteroid and antiviral therapy in the late phase of pregnancy appears safe and should be discussed with patients presenting with pregnancy-associated Bell’s palsy.^67^

As Bell’s palsy has no known cause, multiple treatments have been proposed. However, here we will focus on the most used treatments: corticosteroid therapy, aiming at the anti-inflammatory action of corticosteroids, and antiviral therapy, based on the hypothesis of viral influence, especially the herpes virus, on the pathophysiology of Bell’s palsy. There is evidence that medical treatment is more effective if initiated within 72 h of symptom onset.^81^

#### Corticosteroid therapy

An increase in the rate and level of recovery has been demonstrated among patients receiving early corticosteroid therapy compared with placebo. Recent AAO-HNS guidelines suggest initiating treatment with systemic corticosteroids within three days of symptom onset, for a 10-day course.^78^

A Cochrane systematic review,^82^ published in 2016, confirmed that corticosteroids effectively reduced the number of people with incomplete recovery at six months’ follow-up compared with placebo (Risk Ratio [RR] 0.63, 95% CI 0.5–0.8), with high-quality evidence (GRADE).^83^ This evidence was based on data from seven randomized clinical trials involving 895 participants with Bell’s palsy of varying degrees of severity. Data from three studies (485 participants) showed clearly that people who received corticosteroids developed less motor synkinesis (unwanted facial movements) and crocodile tears (watery eyes while eating or drinking) compared with people who received placebo alone. This finding was based on moderate-quality evidence.^82^

The trials that contributed most to the review used oral corticosteroid therapy for 10 days with at least five days at a high dose (either prednisolone 50 mg, for 10 days or prednisone 60 mg, for five days, then tapered over five days), started within 72 h of symptom onset.^78^, ^81^, ^84^

Considering the risks involved in the administration of high doses for long periods and that in Bell’s palsy the treatment goal is the anti-inflammatory effect of the corticosteroid, very high doses and prolonged dosages are not necessary. Patients who have associated clinical conditions that contraindicate corticosteroid therapy deserve special attention.

The use of corticosteroids in children remains controversial in the literature. Several recommendations have been extrapolated from studies in the adult population. It is inferred that starting treatment within three days of the onset of facial palsy increases the chance of recovery, reduces time to recovery, and decreases the chance of synkinesis. Some authors suggest that corticosteroid therapy should be initiated within seven days of PFP presentation.^4^, ^5^, ^72^, ^85^ Currently, in addition to treating the underlying cause, prednisone or prednisolone (1 mg/kg, for 10 days) can be combined.^4^, ^85^, ^86^

When prescribing corticosteroids during pregnancy, both maternal and fetal health are of concern, especially in the first trimester of pregnancy. This is due to the increased risk of cleft palate, low birth weight, and preterm birth. This relationship, described in the past, has been reassessed in recent studies that have shown that the quality of evidence is very low.^87^, ^88^ Maternal risks of corticosteroid use are similar to those of non-pregnant patients, including hyperglycemia, hypertension, osteoporosis, and increased risk of infection.^89^ However, it remains unclear whether the unfavorable outcome is due to factors intrinsic to pregnancy or the lower rates of medical treatment in this population. What is clear is that these patients are less likely to receive early corticosteroid therapy, with delay or non-institution of well-established therapies, resulting in worse outcomes.^61^, ^67^ This results from the fear of using corticosteroids in the pregnant population, which highlights the importance of an up-to-date approach for these cases.

According to the AAO-HNS guidelines, corticosteroids should be offered at the beginning of treatment with individualized counseling of the pregnant patient.^78^ Women with comorbid conditions that may be aggravated by corticosteroid therapy, such as poorly controlled diabetes mellitus, mental health problems, or hypertension, should be counseled about management and potential side effects. Maternal monitoring should include blood glucose levels, blood pressure, weight, and screening for infections, dyspepsia, and sleep/mood disorders during treatment.

#### Antiviral therapy

Antiviral monotherapy is not recommended, with several studies supporting the recommendation against this practice in Bell’s palsy.^81^, ^84^

When a combination of antivirals and corticosteroids is used, some isolated studies have shown controversial results. However, considering the results of the highest quality and most complete Cochrane systematic review published in 2019, including three clinical trials involving 766 participants, there was no evidence of benefit of antivirals plus corticosteroids for incomplete recovery with a follow-up of three to 12 months (RR = 0.81, 95% CI 0.38–1.74), with imprecise results and low-certainty evidence, suggesting that there may be little or no difference between the combination of antivirals and corticosteroids and corticosteroids alone.^90^ The combination of antivirals and corticosteroids probably reduced the late sequelae of Bell’s palsy (synkinesis) compared with corticosteroids alone (RR = 0.56, 95% CI 0.36–0.87), based on two clinical trials involving 469 patients, with moderate-certainty evidence by the GRADE assessment.^83^, ^90^

Antivirals can be used in cases of a diagnosis of probable Bell’s palsy given the assumption that its cause may be related to viral reactivation, as well as in Ramsay Hunt syndrome. Treatment options include valacyclovir at a dose of 20–30 mg/kg, thrice a day, for 5 days.^4^, ^85^ Acyclovir can be used at a dose of 40–80 mg/kg/day, thrice a day, for 7 days, in children under 12 years of age. In those over 12 years of age, acyclovir can be used at a dose of 200 mg, five times a day, or 400 mg, thrice a day, for 7 days, with a maximum daily dose of 1000 mg/day.^85^

Acyclovir is administered at a dose of 800 mg, five times a day, and has a lower bioavailability than valacyclovir (a prodrug of acyclovir). Valacyclovir has been shown to be superior in the treatment of HZ, with a more comfortable dosage (1 g, thrice a day), but at a higher cost, which should be considered in decision-making.^90^ Both acyclovir and valacyclovir are approved for use during pregnancy, even in cases of early pregnancy, in the treatment of genital herpes, with safety grade B.^91^

Combined corticosteroid/antiviral therapy still lacks clear evidence of significant additional benefit and may be considered in specific cases, especially when viral etiology is likely, also considering that the risk of adverse effects of oral antiviral therapy is very low.^78^, ^90^

#### Additional care

Bell’s palsy can limit the patient’s ability to blink, which may lead to eye pain, irritation, and dryness and, in rare cases, to permanent corneal damage and vision problems. For patients with Bell’s palsy with incomplete eye closure, treatments include frequent daytime use of lubricating eye drops and, while sleeping, use of lubricating eye ointment and/or eye taping or patching to ensure eye closure.^4^, ^71^, ^78^, ^85^^,^^92^ At night, the eye should be covered with a medical grade adhesive after gently applying an ophthalmic ointment.^4^, ^85^, ^92^ All patients with Bell’s palsy should be evaluated for inadequate closure of the eyelid and risk of corneal exposure and abrasion. Patients should be examined for lagophthalmos in both the upright and supine positions and should be reviewed monthly for the first three months to screen for possible corneal exposure. Eye care should be provided to all patients with inadequate eyelid closure early in the course of the disease to prevent the risk of corneal exposure, taking into account the puerperal stage and needs of the patient. If the eyelid still does not close completely after recovery from Bell’s palsy, surgical procedures should be considered, and an ophthalmologic evaluation is suggested.

##### Physical therapy

Given the higher rate of incomplete recovery and poorer long-term outcomes for pregnancy-associated Bell’s palsy, a multidisciplinary team approach is imperative for the comprehensive physical and psychological care of these patients.^93^, ^94^ Physical therapy in Bell’s palsy is focused on preventing and managing the sequelae of incomplete recovery, most commonly synkinesis, hypertonicity, and residual weakness.^95^, ^96^ The value of early physical therapy intervention within a multidisciplinary framework lies in education, appropriate exercises, and follow-up to monitor facial motor recovery.

Most of the patient’s rehabilitation will take place in the postnatal period. Ensuring that the dosage and frequency of exercises respect this period and sleep deprivation is essential. On the affected side, facial exercise techniques are directed at developing motor relearning patterns and training to avoid unwanted movements. On the unaffected side, training aims at avoiding exaggerated movements from an early stage, which can help reduce asymmetry. Improving the subtlety of movement on the unaffected side can help prepare for the eventual return of contralateral movement.

The combination of facial exercises and botulinum toxin therapy has demonstrated benefits for both function and quality of life.^95^, ^96^, ^97^ In the long term, matching the unaffected side to the degree of movement available on the palsy-affected area can balance out movement and aging over time.

##### Psychosocial management

Facial palsy has a great impact on patients’ quality of life and socialization, leading to serious psychological and social problems. Although complete recovery occurs in most cases, those with sequelae or incomplete recovery should be evaluated for the possibility of being offered surgical treatment or other procedures to reduce the aesthetic impact with rehabilitation options.

Currently, there is a paucity of evidence in the literature on the specific benefit of psychotherapy for pregnancy-associated Bell’s palsy. However, it is known that pregnancy and the postpartum period can be a very stressful period. Pregnant patients have been shown to be vulnerable to stress and psychological distress. The development of facial palsy in this setting may worsen psychological outcomes and predispose pregnant patients to increased puerperal psychological distress.

It is well known that rates of depression and psychological distress are significant during pregnancy, particularly in the second and third trimesters, and during the peripartum period.^98^ Facial disfigurement and loss of key facial functions with Bell’s palsy have been associated with significant levels of psychological distress, anxiety, and depression.^99^, ^100^ Although no specialized therapy has been developed specifically for the socioemotional effects of facial palsy, pregnant patients with Bell’s palsy may represent a group that would benefit from early psychotherapist referral.^101^ These patients should certainly receive early psychological and emotional support.

Bell’s PFP during pregnancy and the peripartum period can cause significant functional and psychosocial distress for the patient. During this period, pregnant women are less likely to receive early medical therapy and are at increased risk of incomplete recovery and poorer long-term outcomes. Updated knowledge aims at early treatment and adequate management in these cases. Diagnostic imaging is generally not required for the diagnosis of Bell’s palsy, but if well indicated, it should not be withheld from the pregnant patient after appropriate counseling on the risks involved. Although traditionally less prescribed in the pregnant population, pregnant patients with Bell’s palsy should be offered early corticosteroid therapy. No clear consensus exists as to the benefit of adding antiviral agents to corticosteroids in the acute setting. Antiviral therapy may be offered to pregnant patients, with some benefit seen in patients with complete facial palsy (Table 10).

**Table 10:** Summary of medical management pregnancy-associated Bell’s palsy.

Therapy	When to implement?	Regimen
Oral corticosteroids	Within 72-hs of symptom onset	Prednisone 50 mg daily for 10-days
		Prednisone 60 mg daily for 5-days, then tapered over 5-days
Oral antivirals	Within 72-hs of symptom onset	Valacyclovir 100 mg 8/8 h for 7-days
Protective eye care	Throughout symptomatic course	Daytime: preservative-free eye drops, to the affected eye, hourly
		At night: ointment formulation of artificial tears to the affected eye + eyelid taping with moisture chamber
		Surgically placed external eyelid weights should be considered for complete flaccid paralysis without improvement after 8–10 weeks

It is important to inform the patient of the safety profile of antiviral agents and the expected modest gain in benefit. All patients with inadequate eyelid closure should receive topical eye care to avoid lagophthalmos-associated exposure keratopathy. A multidisciplinary approach is critical in the optimal management of this complex and distressing disorder, with early involvement of physical therapy and psychotherapy and prompt referral to a facial nerve specialist.

### Recommendations

I – Laboratory testing, imaging studies, and electrophysiologic testing have limited indication in the setting of Bell’s palsy and should not be indicated routinely or in cases of incomplete palsy (Strong recommendation; Low-quality evidence).

II – The tests can be performed in specific cases, guided by history-taking and physical examination, as well as in cases of complete palsy or with unfavorable outcome (no signs of improvement after 2–3 weeks, worsening of the degree of paralysis or complete/bilateral/recurrent facial palsy, which may be related to another etiology) (Weak recommendation; Low-quality evidence).

III – Corticosteroid therapy should be encouraged as it reduces the chances of incomplete recovery from Bell’s palsy and the chances of late sequelae (e.g., synkinesis) (Strong recommendation; High-quality evidence).

IV – Combined corticosteroid and antiviral therapy, in cases of incomplete recovery, has little or no effect on motor recovery (Strong recommendation; Low-quality evidence).

V – Antivirals plus corticosteroids have a better effect on the recovery of late sequelae (e.g., synkinesis or lagophthalmos) than corticosteroids alone (Strong recommendation; Moderate-quality evidence).

## Ramsay hunt syndrome

James Ramsay Hunt (1872–1937) described three syndromes. The best acknowledged one is zoster *oticus* with PFP. The second Ramsay Hunt syndrome encompasses the clinical features produced by carotid artery occlusion. The third Ramsay Hunt syndrome was called “*dyssynergia cerebellaris progressiva*”, currently known as spinocerebellar degeneration. Hunt’s research on herpetic inflammation of the geniculate ganglion described, for the first time, the relationship between the geniculate ganglion and sensory function of the facial nerve.^102^

Although Ramsay Hunt syndrome is traditionally defined as zoster *oticus* and PFP, Hunt noted other signs and symptoms such as tinnitus, hearing loss, nausea, vomiting, vertigo, and nystagmus. He explained these features by the close proximity of the geniculate ganglion to CN VIII. The involvement of other cranial nerves, such as V, X, IX, and even others described, III, XI, and XII, and the cervical nerves C2, C3, and C4, is explained by axonal propagation and the vasa nervorum to VZV.^102^, ^103^, ^104^ Maximal facial palsy occurs within one week of symptom onset.^105^

VZV is a virus of the herpes family. It occurs worldwide with no seasonal variations in incidence. The primary infection that results in HZ is caused by the reactivation of latent VZV. The incidence of HZ is age-dependent and ranges from 1.2 to 3.4 per 1000 person-years among younger adults to 3.9–11.8 per 1000 person-years in older patients (over 65 years). The cumulative incidence was estimated at 2.9–19.5 cases per 1000 population from 2002 to 2018, with female predominance.^106^

The incidence of Ramsay Hunt syndrome is approximately 5 cases per 100,000 annually (in the USA population). It is a rare manifestation of VZV. Peitersen evaluated 2570 patients with idiopathic PFP.^59^ There were 116 cases of Ramsay Hunt syndrome. The annual incidence was 2.2 per 100,000, approximately 4.5% of all PFPs. Incidence estimates based on mostly retrospective single-center studies range from 4% to 12% of all PFP cases.^104^ The incidence is higher in patients over 60 years of age.^107^ The condition is less frequent and less severe in children.^102^

### Zoster sine herpete (ZSH)

ZSH is defined as neuropathic pain with virologic evidence of VZV infection. It was originally described by the presence of VZV DNA in the CSF of two patients with chronic radicular pain, without rash, who responded clinically to acyclovir treatment.^108^ ZSH is likely underreported and underestimated. It may be the cause of neuropathic pain of unknown origin. The counterpart among patients with Ramsay Hunt syndrome is characterized by PFP without herpetic vesicles, with the presence of either a 4-fold increase in anti-VZV antibody levels or the detection of VZV DNA in the skin, mononuclear blood cells, or middle ear fluid. The incidence of ZSH varies across studies, depending on the criteria used to detect VZV. In a study of 1705 patients with facial palsy without vesicles, approximately 2.4% had ZSH.^107^

### Diagnosis

Diagnosis is clinical and should be made accurately and quickly to deliver prompt treatment and obtain the best possible outcome. Although the appearance of vesicles usually precedes or presents simultaneously with facial palsy, it may occur later, making the clinical picture initially indistinguishable from Bell’s palsy. Symptoms and signs of vestibulocochlear dysfunction are not always present, but may include hearing loss, tinnitus, vertigo, nystagmus, nausea, and vomiting.

Serum VZV antibody titers are only useful when comparing the acute and convalescent stages of the disease. The use of PCR to detect VZV from the skin of the affected area of the ear may help distinguish between patients with Bell’s palsy and those with early-stage Ramsay Hunt syndrome.^109^

Significant factors for increased risk of poor prognosis:^102^ ENoG with more than 90% demyelination; Facial palsy with HB grade IV or more; Age over 50 years.

Patients with Ramsay Hunt syndrome recover worse than those with Bell’s palsy. But this difference may not be as great as we have thought. Approximately 30% of patients with Bell’s palsy, even with corticosteroid therapy, have some degree of sequelae, which are usually mild.^84^

Herpetic vesicles are most commonly found in the ear (76%), followed by the oral cavity (13%), but they may also occur in other parts of the head and neck (11%).^104^ Time to the appearance of vesicles is variable. It is very important to advise patients to be alert for the appearance of vesicles. Kanerva et al.^104^ reported that vesicles preceded facial palsy in 48% of cases, were concomitant in 16%, and followed the palsy onset in 36%. In the study by Aizawa et al.,^110^ vesicles preceded palsy in 31% of cases, were concomitant in 52%, and followed the palsy onset in 15%, being observed from 27 days before to 13 days after the palsy onset. They investigated the patients’ viral load and immune responses and concluded that facial palsy in Ramsay Hunt syndrome can occur at different times, from early phase to the regression phase of VZV reactivation, and that there are variable patterns of development of facial nerve dysfunction induced by VZV reactivation with progression of neuritis.^110^

Studies have shown variable presentations of hearing loss associated with Ramsay Hunt syndrome (20%–70% of cases).^59^, ^104^ The prognosis for sensorineural hearing loss is good, only 5% of patients will have residual hearing impairment.^109^ Other potentially chronic sequelae include post-herpetic neuralgia, tinnitus, and vestibular dysfunction.^111^

### Treatment

#### Antiviral therapy

The first-line treatment for HZ infections is acyclovir, which can be administered intravenously or orally. Other antivirals may also be prescribed – valacyclovir, famciclovir, or brivudine. Antiviral therapy is most effective when started within 72 h of symptom onset.^111^ If the diagnosis is made and antiviral therapy is started within this time frame, the rate of recovery from facial palsy may reach up to 80% of patients, according to non-randomized studies.^104^, ^109^ Before the use of antivirals or combined therapy, poor PFP recovery results were reported (30%–46%).^59^, ^112^

Antiviral therapy is effective against viral replication in HZ infections. It can prevent further proliferation and spread of VZV, but it cannot eliminate the virus. Oral antiviral therapy has been used to treat HZ infections in other parts of the body. The use of these agents reduces the severity of HZ infection in immunocompetent adults, the duration of viral shedding, and new lesion formation,^113^ thereby accelerating rash healing and reducing the duration of pain.^114^

Oral antivirals should be used for seven to 10 days, starting within 72 h of the onset of a rash. They are generally very well tolerated if administered at standard doses (acyclovir: 800 mg, five times a day, for 7–10 days; famciclovir: 500 mg, thrice a day, for s7 days; valacyclovir: 1000 mg, thrice a day, for 7 days).^113^ Oral brivudine appears to have a higher potency against VZV than other oral medications. It also has a simpler regimen (once a day) and no nephrotoxic effects. Patients should remain well hydrated during treatment. The most common side effects are nausea (rarely vomiting) and headache, which occur in 10%–20% of cases. Other possible side effects from prolonged use include impaired kidney function, diarrhea, dizziness, fatigue, skin rash, anorexia, leg pain, sore throat, and hair loss.^111^

Antivirals cannot prevent the acute or chronic pain caused by the infection, which occurs in 20% of patients over 50 years of age. Therefore, corticosteroids have been used to reduce nerve inflammation and associated pain.^115^

#### Corticosteroid therapy

Corticosteroids are used in a wide variety of conditions. In HZ infections, synthetic glucocorticoids such as prednisone and prednisolone are often prescribed for their anti-inflammatory properties. Two clinical trials demonstrated that the use of corticosteroids for 3 weeks did not contribute significantly, beyond the benefits achieved by acyclovir alone, to better PFP outcomes in Ramsay Hunt syndrome. Some authors indicate the addition of corticosteroids for relief of local pain beyond that achieved with antiviral therapy alone.^111^

Corticosteroids should be considered for patients with VZV-induced facial palsy with polyneuritis, peripheral nerve damage from foraminal compression, or evidence of central nervous system involvement. Contraindications (hypertension, diabetes, gastritis, osteoporosis, and psychosis) and risks associated with the use of corticosteroids must be carefully evaluated.^116^

#### Vaccination

Two vaccines are currently available – a live attenuated VZV vaccine or “Live Zoster Vaccine” (LZV) and an adjuvanted recombinant subunit zoster vaccine or “Recombinant Zoster Vaccine” (RZV). LZV has been the standard vaccine for years. The safety and efficacy of both vaccines have been demonstrated in clinical trials in healthy immunocompetent adults, in selected immunocompromised patients, and in patients with immune disorders. RZV is more effective in preventing HZ than LZV. RZV is non-replicating and therefore safe also for immunocompromised persons.^117^ They have been effective in preventing HZ disease for up to three years (the main studies have not followed participants for more than three years). Both vaccines produce systemic and injection site adverse events of mild to moderate intensity.^118^

A Chinese study analyzed the potential public health impact of RZV, compared with no vaccination, in individuals aged 50 years and older. Mass vaccination with RZV was estimated to prevent more than 430,000 cases of HZ and more than 51,000 cases of post-herpetic neuralgia compared with no vaccination. The authors suggested that more than 14,000 hospitalizations and more than 1,000,000 outpatient visits could be avoided. Patients aged 50–59 years had the greatest overall reduction in HZ cases, its complications, and related health care resource utilization.^119^

### Recommendations

I – Ramsay Hunt syndrome is a rare condition. Patients with PFP should have the oral cavity and ear carefully inspected for herpetiform skin lesions that indicate VZV infection (Strong recommendation; Moderate-quality evidence).

II – Patients with PFP, without vesicles and with severe otalgia, should undergo a differential diagnosis with ZSH and initiate treatment (Strong recommendation; High-quality evidence).

III – Treatment should be started as early as possible (within 72 h of onset) (Strong recommendation; High-quality evidence).

IV – In Ramsay Hunt syndrome, 7-day corticosteroid therapy with prednisolone or methylprednisolone combined with antiviral therapy should be started as soon as possible (Strong recommendation; High-quality evidence).

V – Vaccination has reduced the incidence of HZ infections for at least three years (Strong recommendation; High-quality evidence).

## Facial palsy after temporal bone fracture

PFP secondary to temporal bone fracture, before being considered an otolaryngologic condition, is the result of high-energy head trauma, and therefore should be initially managed as a medical emergency that can lead to patient death. In the initial approach, it is imperative to follow the Advanced Trauma Life Support (ATLS) guidelines so that potentially fatal causes of trauma are excluded, and the patient can then undergo a secondary assessment.^120^

Injuries secondary to temporal bone fracture of interest to the otolaryngologist include damage to the otic capsule, vestibulocochlear nerve, ossicular chain, tympanic membrane, external auditory canal, temporomandibular joint, and adjacent vessels (jugular vein and carotid artery), CSF leak, and finally, damage to the facial nerve, which presents as traumatic PFP.^121^ The medical-surgical management of traumatic PFP is both challenging and controversial.^122^

The most common cause of congenital PFP is perinatal trauma, typically presenting as unilateral facial palsy and associated with favorable prognosis. Risk factors include primiparity, prematurity, forceps delivery, and macrosomia.^3^ The embryological development of the facial nerve is associated with the second branchial arch, which explains the association of PFP with congenital malformations of the first and second branchial clefts, such as hemifacial microsomia.^3^ Regarding pediatric trauma, a unilateral presentation is often observed, but blunt trauma may cause extensive fractures, leading to bilateral facial palsy.^3^ Penetrating injury to the face may cause motor deficits corresponding to the injured facial nerve branches.^3^

The dilemma faced by the otolaryngologist in this situation is whether facial nerve decompression is indicated or not and what is the optimal timing for decompressing it.

### Radiologic diagnosis

Traditionally, temporal bone fractures are classified by CT as longitudinal, transverse, or mixed relative to the petrous ridge. This classification was first described by Ulrich, in 1926.^123^ Longitudinal fractures account for 70%–80% of cases. Transverse fractures are less frequent, accounting for 10%–20% of cases, and mixed fractures occur in 10%.^124^, ^125^

Diaz et al., however, proposed a new classification based on the involvement of the otic capsule by the fracture, regardless of the direction of the fracture line. It is divided into Otic Capsule Disrupting (OCD) and Otic Capsule Sparing (OCS) fractures.^121^ This classification is important because it allows predicting the severity of the signs and symptoms that the patient may develop. In OCD, there is involvement of the otic capsule with damage to adjacent structures, including the facial nerve. Compared with Ulrich’s classification, these injuries to the otic capsule are usually more closely associated with transverse fractures, but they may also be present in longitudinal fractures at a lower incidence. In OCS, the otic capsule is spared. This translates into a lesion with less potential for injury to the structures of the inner ear and facial nerve. Likewise, fractures classified as longitudinal are often associated with mild injury, but in 20% of cases the fracture line, although longitudinal, may disrupt the otic capsule.^121^, ^126^

### Clinical classification

The risk of developing traumatic PFP is approximately 6%–7% for patients with temporal bone fractures. Of these, 25% are an acute, complete paralysis already present at the trauma scene. The remaining 75% are partial at the time of initial assessment with progressive deterioration over the days following the injury.^121^, ^123^, ^126^, ^127^

Treatment of traumatic PFP is controversial, and the importance of differentiating between these two forms of presentation lies in the different prognosis and, subsequently, different treatment options.^121^, ^126^ Patients who have acute, complete facial palsy (at the trauma scene) often develop a higher degree of axonal injury (Sunderland classification), with a potential for non-regeneration of the nerve fiber. Patients who have a milder degree of PFP at onset often develop a milder degree of axonal injury, with a high potential for regeneration. Both groups should receive corticosteroid therapy (oral or parenteral) within the first 24 h and be routinely evaluated within the first two weeks.^126^, ^128^

In pediatric patients presenting with traumatic brain injury, after clinical stabilization and care according to the ATLS guidelines, temporal bone CT without intravenous contrast is indicated for proper imaging of the entire course of the facial nerve for prognostic evaluation and medical-surgical treatment.^5^ Regarding the trauma, a unilateral presentation is often observed, but blunt trauma may cause extensive fractures, leading to bilateral PFP.^3^ Penetrating injury to the face may cause motor deficits corresponding to the injured facial nerve branches.^3^

### Indications for surgical decompression of the facial nerve

The choice of approach for the previously mentioned groups (considering clinical classification), that is, either the maintenance of medical treatment or the decision to surgically decompress the facial nerve, must be made during the two weeks of clinical follow-up. The first group will likely show, after 14 days of corticosteroid therapy, poor recovery of facial nerve function. In these cases, NCS and EMG are important to assess nerve viability.^121^, ^126^, ^128^ These tests should ideally be performed 10–14 days post-injury, when Wallerian degeneration has taken place. When the examination shows a degree of degeneration greater than 90% within 14 days of injury, a potentially unrecoverable Sunderland degree V injury may have occurred.^121^, ^126^ Facial nerve decompression is indicated in these cases.

The optimal timing for decompression is as early as possible. Studies have demonstrated that the best outcomes are obtained with decompression performed within two weeks of the onset of the palsy.^129^, ^130^ Considering the clinical status of the patient (who may have other medical-surgical priorities due to the trauma), an early approach may be a difficult task. In these cases, some studies have reported that decompression may be beneficial even if performed within two months of the event.^129^, ^130^, ^131^ Surgical treatment in the pediatric population is indicated in cases of post-traumatic PFP of immediate onset, with surgical exploration and suture or nerve decompression being indicated, depending on the intraoperative findings.^5^

### Recommendations

I – Patients with temporal bone fracture leading to PFP should be initially approached by quantifying the degree of involvement using the HB grading system (or any other scale mostly used in the center) and temporal bone CT imaging (thin-slice CT scans) (Weak recommendation; Low-quality evidence).

II –Electrophysiologic testing of the facial nerve, in cases of complete facial palsy, can be performed 10–14 days after the onset of the palsy (Weak recommendation; Low-quality evidence).

III – Medical treatment is performed with oral or intravenous corticosteroid therapy, depending on the patient’s neurologic status (Weak recommendation; Low-quality evidence).

IV – Surgical decompression of the facial nerve is suggested when electrophysiologic testing shows degeneration greater than 90%. The optimal timing for decompression is within two weeks of the onset of the palsy (Weak recommendation; Low-quality evidence).

## Recurrent facial palsy

Recurrent facial palsy is a rare condition that affects approximately 3 per 100,000 patients per year. The overall prevalence of recurrence of an episode of facial palsy ranges from 2.6% to 11%, with most studies reporting rates of 5%–7%.^132^, ^133^, ^134^ Recurrent facial palsy has a positive family history in about 20% of cases, suggesting a genetic predisposition.^133^ There is no evidence of laterality predominance or difference between ipsilateral and contralateral recurrences in idiopathic cases.^134^ Regarding recurrent facial palsy in children, its prevalence is estimated at 4.8%–11% of cases.^71^, ^72^ Most cases are considered idiopathic, but some authors associate its occurrence with sequential viral reactivation, Melkersson–Rosenthal syndrome, and autoimmune diseases.^71^

### Diagnosis

The investigation is based on clinical examination, MRI, CT, chest X-Ray, serology (HSV, VZV, and Human T-Lymphotropic Virus 1 [HTLV-1]), inflammatory tests, and syphilis screening. In rare cases, CSF analysis is warranted to investigate meningeal metastases.^132^ The clinical conditions most associated with recurrent facial palsy are diabetes mellitus, hypertension, and pregnancy.^132^, ^133^, ^134^

The main causes of recurrent facial palsy are Bell’s palsy (idiopathic), with a prevalence of 77%–84%, and Melkersson–Rosenthal syndrome, accounting for 7%–10% of cases. Less frequently, it can be caused by tumors (schwannoma, 3%), Ramsay Hunt syndrome (2%), otitis media, multiple sclerosis, sarcoidosis (3%), Crohn’s disease, amyloidosis, and autoimmune diseases (2%) such as vasculitis with polyangiitis and Sjögren syndrome.^135^, ^136^, ^137^ There are also rare reports of celiac disease, facial baroparesis (scuba diving or flying), and migraine with cranial nerve involvement, similar to ophthalmoplegic migraine.^138^

**Figure 6:**
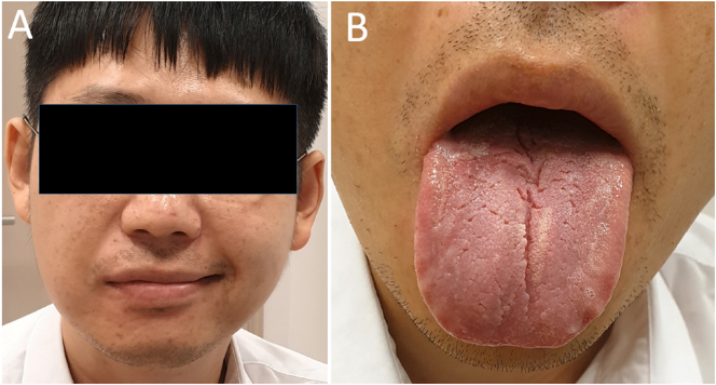
Characteristics of Melkersson–Rosenthal Syndrome. Facial paralysis in (A) and lingua plicata in (B).

Melkersson–Rosenthal syndrome is characterized by recurrent orofacial edema, recurrent PFP, and fissured tongue (Fig. 6).^139^ The etiology is unknown with an autosomal dominant inheritance with variable expressivity. The diagnosis is essentially clinical.^137^ Lip biopsy may show lymphomonocytic infiltration, non-caseating epithelioid granulomas, Langerhans-type multinucleated giant cells, and fibrosis. The incidence of Melkersson–Rosenthal syndrome with facial palsy is 0.36 per 100,000 patient-years, and 75% of patients have at least one recurrence.

The recovery rate from recurrent facial palsy is lower than that from a primary episode.^135^ Chung et al.^134^ found 72.2% of functional recovery (HB grade I or II) in patients with recurrent Bell’s palsy vs. 88.4% in those with primary Bell’s palsy. The recovery rate seems to worsen with each recurrence. This is possibly due to neural desynchronization resulting from poor myelination and small diameter fibers of regenerated axons. Furthermore, the risk of recurrence increases with each new episode, ranging from 15% after the second event to 50% after the fourth event.^140^ The recovery rate in Melkersson–Rosenthal syndrome is much lower than in cases of recurrent Bell’s palsy (13% vs. 71%), leading to a much worse prognosis.^139^ There are no significant differences in the presentation of recurrent facial palsy between children and adults.^141^ The best prognostic factors are initiation of treatment within seven days and favorable EMG results.^134^

Electrophysiologic testing has a limited role, as sequelae from previous episodes may affect action potentials. Yoshihara et al. showed that ENoG was not significant in the assessment of functional recovery rates. Surface EMG with stimulation on the mandibular region proved to be an effective parameter for prognostic evaluation when the interval between episodes was greater than four years.^135^

### Treatment

Recurrent Bell’s palsy requires the same clinical management as the primary episode. Early corticosteroid treatment may be initiated in the presence of prodromal symptoms.^133^

Surgical decompression of the facial nerve aims to relieve neural inflammatory edema to reduce functional deterioration. It also aims to prevent or reduce the number of subsequent episodes.^140^ Total decompression of the facial nerve (intracanalicular, labyrinthine, geniculate ganglion, tympanic, and mastoid segments – via middle cranial fossa combined with transmastoid approach) for Melkersson–Rosenthal syndrome was evaluated in a non-randomized clinical trial, compared with conventional medical treatment. Surgery was performed within the first 21 days of onset and reduced from 50% to 0% (*p* < 0.05) the recurrence rate in the syndrome compared with the control group during a 5-year follow-up. Furthermore, the recovery rate (HB grade I or II) was 89% vs. 66% (*p* > 0.05), with apparent superiority of surgical treatment.^142^ Decompression only via middle cranial fossa approach (IAC, *porus acusticus internus*, labyrinthine segment, and geniculate ganglion) for recurrent Bell’s palsy was evaluated in a retrospective cohort study with good results.^143^ The recurrence rate was 8.3% in the surgery group vs. 64% in the control group (*p* < 0.05). The recovery rate (HB grade I or II) was 91% vs. 50%. Subtotal facial nerve decompression via transmastoid approach (from the geniculate ganglion to the stylomastoid foramen) reduced recurrence in patients with Melkersson–Rosenthal syndrome in a case series.^144^

### Recommendations

I – Recurrent facial palsy, although rare, deserves special attention from the physician. Its progression compromises the quality of life of affected individuals and gradually worsens nerve function. Detailed etiologic investigation and individualized treatment are essential. Imaging is suggested (MRI and CT) to exclude other conditions associated with recurrent facial palsy, such as otitis media, spreading lesions, and trauma (Moderate recommendation; Moderate-quality evidence).

II – Complementary tests, such as chest X-Ray, serology (HSV or VZV), inflammatory tests, syphilis screening, and fasting glucose test, are suggested for investigation (Weak recommendation; Low-quality evidence).

III – HTLV-1 serology and CSF analysis are suggested to investigate meningeal metastases (Weak recommendation; Low-quality evidence).

IV – Clinical treatment follows the recommendations for the treatment of the primary episode, that is, the use of corticosteroids and antivirals is suggested (in cases clearly associated viral infection) (Strong recommendation; Low-quality evidence).

V – Surgical decompression of all segments of the facial nerve, via middle cranial fossa and transmastoid approaches, is suggested (Strong recommendation, Low-quality evidence).

VI – Subtotal facial nerve decompression, only via middle cranial fossa approach, may also be a surgical option (Moderate recommendation; Moderate-quality evidence).

VII – However, subtotal facial nerve decompression from the geniculate ganglion to the stylomastoid foramen, via transmastoid approach, is rarely suggested (Weak recommendation; Low-quality evidence).

## Facial nerve tumors

Primary tumors of the facial nerve are rare lesions that can affect any facial nerve segment (intracranial, intratemporal, and extratemporal).^145^, ^146^ Schwannomas are the most common tumors. Other less frequent tumors include neurofibromas, geniculate ganglion hemangiomas, and granular cell tumors.^145^ The diagnosis of these tumors can be quite challenging given their intimate anatomic relationship with other structures of the lateral skull base. The clinical course is also quite variable, as it depends on tumor location, with clinical symptoms like those of other more frequent lesions that can indirectly affect the facial nerve.^145^

The treatment of facial nerve tumors is also challenging because of the high risk of facial nerve injury in any surgical procedure. The treatment initially proposed and robustly used for decades is total resection of the tumor with nerve grafting, a procedure that results in HB grade III in patients with better outcomes.^146^ Considering the high variability of degrees of facial palsy that patients present at the time of diagnosis, surgical intervention, in most cases, may lead to further worsening of nerve function. Over the years, the treatment paradigm has become more conservative. This has occurred mainly because of the observation that facial nerve tumors are mostly benign and grow slowly, which may obviate the need for surgical treatment.^147^ Therefore, it is necessary to make prudent decisions, especially in young patients and those with minimal facial paralysis.^146^ Given the rarity of these lesions, it is difficult to develop a clear consensus regarding the optimal treatment strategy.^145^

Facial nerve schwannomas, although rare, are the most common tumors affecting the facial nerve (64.3% of all intrinsic tumors).^148^ Facial nerve schwannomas represent one of the most complex treatment paradigms in otology/neurotology.^147^ Given their rarity, it is extremely complex to determine their true incidence in the general population. Saito and Baxter^149^ reported a 0.83% prevalence of incidental schwannomas in human temporal bones. However, this rate is not representative of the general population and, therefore, the true incidence of facial nerve schwannomas remains relatively unknown.

### Clinical presentation

Symptoms of facial nerve schwannoma depend on a number of factors, but mostly on tumor location and size. In patients diagnosed with facial nerve schwannoma, the most common symptom is hearing loss, affecting 33%–78.6% of patients.^145^ Other symptoms include tinnitus (7%–51.8%) and vertigo (46%).^147^, ^150^ Specifically regarding the facial nerve, 50%–60% of patients have a history of paralysis, being transient in approximately 20%.^145^

### Histopathology

Like vestibular schwannomas, facial nerve schwannomas arise from Schwann cells and are classified into Antoni A, Antoni B, or combined histologic pattern.^145^, ^151^ Antoni A lesions are characterized by spindle cells with elongated nuclei arranged in a variety of patterns. On cross section, these cylindrical cells produce a palisade over a central mass of cytoplasm called a Verocay body. Antoni B pattern consists of loose connective tissue, lacking the arrangements seen in Antoni A. Antoni B pattern is understood as a degenerative form of Antoni A pattern.^152^ There are no descriptions of a correlation between histologic pattern and tumor behavior.

### Diagnosis

Physical examination of patients may be normal.^147^ Careful examination of the facial nerve is extremely important, particularly to evaluate minor abnormalities (such as tremors, segmental palsy, or other more subtle abnormalities).^145^ Otoscopy is typically normal unless there is a large lesion involving the tympanic or mastoid segment, which may result in a retrotympanic mass or lesion invading the external auditory canal.^153^ In these situations, tuning fork tests can reveal conductive hearing loss on the involved side. Likewise, when there is sensorineural hearing loss (schwannoma involving the IAC or the cerebellopontine angle), the Weber test can lateralize to the unaffected side.

Imaging is essential for the diagnosis and staging of the lesion. MRI is the main diagnostic tool for facial nerve injury. Thin-sectioned contrast-enhanced (gadolinium) MRI usually shows a hyper-uptake lesion along the facial nerve. The most commonly involved segment is the geniculate ganglion (60%–66%), followed by the tympanic (53%) and labyrinthine (50.6%–60%) segments.^147^, ^150^ Most tumors involve more than one segment.^145^, ^147^, ^150^, ^154^, ^155^, ^156^, ^157^ In some cases, several schwannomas may affect the nerve in different locations, appearing as “beads on a string” on MRI scan, which occurs in 20% of cases.^150^, ^159^ When segments proximal and distal to the geniculate ganglion are involved, the typical “hourglass” appearance can be seen on MRI scan. However, differentiating facial nerve schwannomas from vestibular schwannomas can be challenging, if not impossible, when only segments proximal to the labyrinthine portion are involved. Similarly, if only the geniculate ganglion or the tympanic or vertical segments are involved, diagnosis can be complex because these segments can present a hyper-uptake aspect under normal physiologic conditions.

### Treatment

Determining the optimal treatment of patients with facial nerve schwannoma is complex. The main goal of treatment is preservation of facial nerve function for as long as possible, assuming other symptoms do not require intervention. Multiple treatment methods have been proposed in the literature. These include conservative management with periodic imaging surveillance, decompression, tumor debulking, and gross total resection. Many of the lesions that affect solely the intracanalicular segment and/or cerebellopontine angle are initially preoperatively misdiagnosed as vestibular schwannomas until the tumor is evaluated intraoperatively. The rates of misdiagnosis of vestibular schwannoma in cases of facial nerve schwannoma in these locations range from 22% to 64.2%.^145^, ^158^ With improved MRI resolution, this misdiagnosis rate is likely to reduce. Like vestibular schwannomas, facial nerve schwannomas are slow-growing lesions (between 0.85 and 1.4 mm per year).^147^, ^156^ Therefore, most authors recommend the adoption of conservative management with periodic imaging when symptoms are minimal and facial nerve function remains better than HB grade III.^145^, ^147^, ^158^, ^159^ On average, facial nerve schwannomas are followed for more than five years before requiring intervention, and some patients do not need additional treatment. Patients who do not require intervention are often able to maintain their baseline facial nerve function without significant impact on hearing.^145^, ^147^ This outcome, although ideal, is not always possible, especially in cases of rapid tumor growth and worsening symptoms.

Decompression of the facial nerve aims to preserve its integrity and relieve pressure, improving axonal flow.^157^ The results described in the literature show that several patients treated with this technique do not present a significant worsening of facial nerve function over the years.^147^, ^159^ Wilkinson et al.^147^ described 19 patients treated with facial nerve decompression with a mean 6-year follow-up. Four patients (21%) had a decline in nerve function, and three showed improvements in function. These patients also showed a reduction in lesion growth rates (0.17 mm/year vs. 0.85 mm/year in the observational cohort). Therefore, decompression has become a reasonable option in patients with lesions whose growth is confined to the Fallopian canal and with progressive worsening of facial nerve function.

Tumor debulking, although introduced about five decades ago, remains a controversial strategy.^160^ The main argument against debulking is that histologic studies have shown that some facial nerve fibers pass through the tumor and may be damaged by this technique.^161^, ^162^ However, several studies have shown good outcomes using debulking.^145^, ^150^, ^158^, ^159^, ^160^, ^163^, ^164^ One of the factors that has increased the use of debulking is the introduction and improvement of facial nerve monitoring techniques. With monitoring, portions of the tumor that are not directly stimulated can be removed until fibrillation potentials are observed.^150^, ^159^ Unfortunately, monitoring has not proven to be a good predictor of the final postoperative facial nerve function.

Based on tumor size and location, symptoms, and preoperative hearing, debulking of facial nerve schwannomas can be performed either through a middle cranial fossa, transmastoid, or translabyrinthine approach or through a combined approach. Mowry et al.^159^ reported 11 cases of schwannomas of the facial nerve that affected the IAC and/or the cerebellopontine angle separately and underwent debulking. The authors were able to remove 95% of the tumor in nine patients (81.8%), 80% in one patient (9.1%), and 66% in one patient (9.1%). Postoperatively, most patients who had a preoperative HB grade III facial palsy or worse improved their nerve function to a grade I or II, and only one patient had worsening nerve function compared with baseline (changed from HB grade I preoperatively to grade IV postoperatively).

Data on subtotal tumor resection are rare but show that a specific subset of patients can maintain good facial nerve function postoperatively. A study with a mean of seven years of follow-up demonstrated that it was possible to perform 95% tumor resection in 10 patients and 70%–80% resection in five patients.^150^ Overall, 27% of patients experienced tumor growth over time. This rate increased to 60% when considering patients undergoing 70%–80% debulking. Although the sample size was small, there was a clear trend toward increased likelihood of growth in tumors undergoing smaller percentage resections. These data are helpful in counseling patients postoperatively, although they are not effective in the future making-decision process, because it is impossible to know the extent of safe tumor debulking preoperatively.^150^

In patients presenting with poor facial nerve function (HB grade >III), total facial nerve resection with reconstructive techniques is generally recommended. In these cases, studies have shown that preoperative facial nerve function improves or remains the same in 52%–55% of patients, with 84% having a final HB grade III or IV.^145^, ^147^ Particularly difficult cases are those in which the facial nerve is found to be involved at the level of the brainstem, without a proximal stump, and are therefore not amenable to grafting.

In recent years, there has been great variability in approaches related to the treatment of facial nerve schwannomas.^145^, ^147^ Before 1995, 85% of facial nerve schwannomas underwent resection and 15% decompression, with clinical observation not being considered a feasible option.^147^ In contrast, in patients diagnosed after 1995, only 27% underwent resection, 32.7% decompression, and 29% clinical observation. A similar pattern showing a trend toward more conservative management has been demonstrated by researchers at Vanderbilt University. According to the group, the number of resections of facial nerve schwannomas with grafting decreased from 74% to 40%, while the number of subtotal resections increased from 2.1% to 30% during the same period.^145^

A more recent addition to the treatment of schwannomas is the application of stereotactic radiosurgery. Unfortunately, early descriptions of the method’s effectiveness reported vague results for tumor control and postoperative outcomes related to hearing, balance, and nerve function. Recently, standardized outcome measures have been published and become increasingly used. Recent studies have shown tumor control rates ranging from 83.3% to 100%.^145^, ^147^, ^165^, ^166^, ^167^, ^168^, ^169^, ^170^, ^171^ In most patients, facial nerve function has remained unchanged, but there are also several reports of patients with worsening nerve function postoperatively.^147^, ^165^, ^166^, ^171^ One of the main factors that prevent a better understanding of the outcomes of stereotactic radiosurgery for facial nerve schwannomas is that a significant number of patients underwent previous surgical procedures, especially in cases in which the tumors were initially misdiagnosed as vestibular schwannomas. Presumptively, these patients underwent decompression surgery without tumor resection, and, therefore, the results from the stereotactic procedure may not be representative of the technique. Long-term follow-up data are needed to determine the efficacy of this treatment.

### Decision-making process in facial nerve tumors

The decision-making process in patients with facial nerve tumors is quite complex. The main difficulties arise from the fact that facial nerve injuries are rare and commonly have a clinical presentation very similar to that of other more frequent injuries in this topography (e.g., vestibular schwannomas). Regarding treatment, there is an important risk of facial nerve function loss even in approaches considered to be more conservative. Considering that facial nerve tumors are mostly benign and grow slowly, the risks and benefits of surgical procedures must be balanced according to the individualized needs of the patient. Given that facial nerve tumors are quite rare, and the procedures performed are constantly being improved, so far there is no systematic diagnostic and treatment strategy that is considered the gold standard for these lesions.

The first factor to be considered is the degree of facial nerve function impairment. Schwannoma, the most common tumor, has a slow growth rate and can be followed radiologically in combination with symptom surveillance. Overall, tumors that result in mild paralysis (HB grade II) can be followed radiologically, whereas surgical treatment should be proposed when there is HB grade III facial palsy or higher.

**Figure 7:**
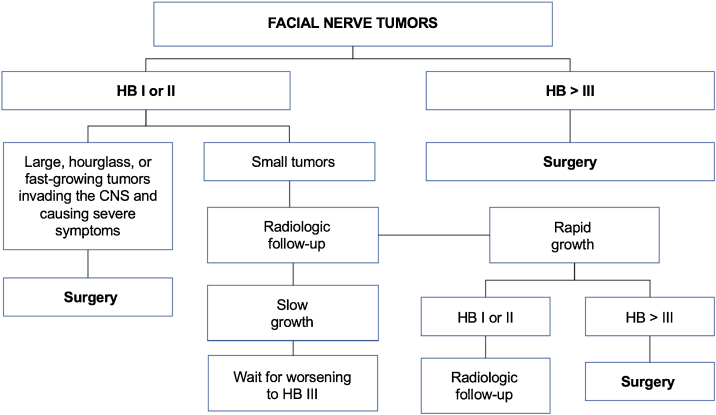
Proposed algorithm for follow-up and treatment of intrinsic facial nerve tumors. Adapted from Prasad et al.

In some cases, early surgical intervention is recommended regardless of the degree of facial palsy: (1) Extratemporal facial nerve tumors extending into the parotid bifurcation (due to reconstruction-related issues); (2) Multisegment facial nerve tumors extending into the cerebellopontine angle and the middle cranial fossa; (3) Fast-growing facial nerve tumors associated with progressive functional impairment; and (4) Large tumors with temporal lobe compression. An algorithm proposed by Prasad et al.^146^ summarizes the main recommendations (Fig. 7).

The surgical treatment to be proposed is still surrounded by great controversy in the literature. Any surgical procedure can result in some degree of immediate postoperative facial nerve palsy. The first decision is whether to perform total or subtotal resection. Total tumor resection is often associated with lower recurrence rates, although facial function is impaired. Subtotal resection, however, could hypothetically result in better functional outcomes, but with higher recurrence rates. Overall, small slow-growing tumors, confined to the temporal bone, are good candidates for subtotal resection.

**Figure 8:**
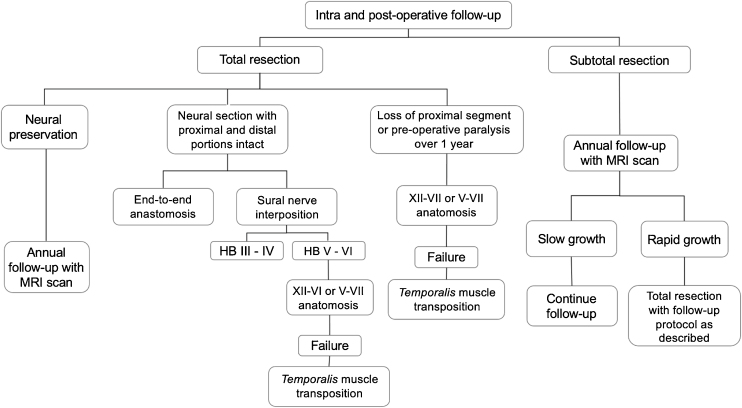
Recommendations regarding intraoperative and postoperative management in the treatment of facial nerve injuries.

A flowchart adapted from Prasad et al.^146^ summarizes the main recommendations regarding intraoperative and postoperative management for facial nerve tumors (Fig. 8).

In summary, facial nerve tumors comprise a wide range of different lesions that can result in facial nerve function loss. Diagnosis is challenging, as many of the symptoms are nonspecific, and facial palsy may develop later in the course of the disease. Given the rarity of these lesions and the additional risk of facial nerve function loss related to surgical procedures, there is great heterogeneity in the diagnostic and treatment approaches for facial nerve tumors. In fact, treatment has changed significantly over the past 2 decades, with more conservative approaches gaining ground over total resection surgery.

### Recommendations

I – Imaging tests to evaluate the IAC and cerebellopontine angle, especially nuclear MRI. The diagnosis of different tumor types involving the facial nerve (e.g., hemangiomas) should be considered in any patient with facial palsy disproportionate to the tumor size (Strong recommendation; High-quality evidence).

II – CT can complement MRI to evaluate changes in the bone architecture around the facial nerve in the tympanic and mastoid segments, typically showing widening of the Fallopian canal compared with the contralateral side. This imaging modality is useful to distinguish between schwannomas and hemangiomas (Weak recommendation; Low-quality evidence).

III – Tumors that result in mild paralysis (HB grade II) can be followed radiologically, whereas surgical treatment may be proposed when there is HB grade III facial palsy or higher, with facial nerve resection and grafting (Moderate recommendation; Low-quality evidence).

IV – Facial nerve decompression is an option in cases of lesions confined to the Fallopian canal and with progressive worsening of motor function (Weak recommendation; Low-quality evidence).

V – Tumor debulking remains a controversial strategy (Weak recommendation; Low-quality evidence).

VI – Application of stereotactic radiosurgery is an option in the treatment of schwannomas, but with vague results for tumor control and postoperative outcomes related to hearing, balance, and nerve function (Weak recommendation; Low-quality evidence).

VII – Treatment of facial nerve tumors can be summarized as follows: small lesions with mild facial paralysis (HB grade II) are followed radiologically, whereas cases with more severe paralysis (HB grade III or higher) are treated via tumor resection either through the middle cranial fossa approach (preserved hearing) or through the translabyrinthine approach (in patients with no serviceable hearing) (Weak recommendation; Low-quality evidence).

VIII – Early surgical intervention is suggested, regardless of the degree of facial palsy, when extratemporal facial nerve tumors extend into the parotid bifurcation, multisegment facial nerve tumors extend into the cerebellopontine angle and the middle cranial fossa, fast-growing facial nerve tumors are associated with progressive functional impairment, and large tumors have temporal lobe compression (Weak recommendation; Low-quality evidence).

## Facial palsy in children

The etiology of facial palsy in children remains unknown in 50% of cases.^3^ Patients with defined etiology are divided into two groups: congenital and acquired, the latter being subdivided into infectious, traumatic, malignancy associated, hypertension associated, and idiopathic.^3^, ^5^

PFP can also be found in syndromic malformations such as syringobulbia, Arnold-Chiari syndrome, and Goldenhar syndrome. Congenital PFP with bilateral presentation, although rare, is most commonly found in Möebius syndrome, with associated palsies of CNs VI, IX, X, and XII.^3^, ^5^ Albers-Schönberg disease is an inherited disorder of bone metabolism, consisting of a gradual increase in bone density, which may cause bone growth along the course of the facial nerve, causing compression and facial nerve palsy, including a bilateral presentation.^5^ Possible etiologic factors also include a group of hereditary myopathies and 3q21-22 and 10q21.3-22.1 gene mutations.^3^

Among potential autoimmune/inflammatory causes, Kawasaki disease and Henoch-Scholein purpura should be considered. Neoplastic causes include facial nerve schwannoma, hemangiomas, leukemia, astrocytoma, rhabdomyosarcoma, and parotid gland tumors.^3^, ^4^, ^5^

Cardiovascular diseases are also associated with PFP in children. The presence of hypertension is considered a risk factor for the development of facial palsy.^5^ It is believed that blood pressure peak leads to hemorrhage in the Fallopian canal, causing autonomic nervous system dysautonomia.^5^ Strokes, although usually presenting with a pattern of central facial palsy, may affect the entire ipsilateral side if the event occurs after the nucleus of the facial nerve.

### Diagnosis

Regarding acquired causes, Lyme disease is among the main causes. Lyme disease is classically divided into three stages. The first stage is known as early localized stage, where the presence of erythema migrans is observed, as well as general symptoms such as malaise, myalgia, arthralgia, low-grade fever, and night sweats. The second stage is known as early disseminated stage and may occur weeks to months after infection, affecting the central nervous system, joints, and heart. The third stage is characterized by late manifestations that may occur months to years after infection, being less frequent, including changes in the skin, central nervous system, and joints.^172^ If Lyme disease is suspected, in addition to thorough physical and neurological examination, the investigation should be complemented with blood and CSF analysis. Studies examining the CSF of pediatric patients with Lyme-attributed PFP showed that 68% had abnormal CSF findings, of whom 55% demonstrated lymphocytic pleocytosis, 45% showed elevated protein, and 33% had evidence of both abnormalities.^5^, ^172^, ^173^ Among these patients whose CSF was examined, 82% did not have antibodies to *B. burgdorferi* in the CSF (Table 11, Table 12).^5^

**Table 11:** Comparison of cerebrospinal fluid analysis results between patients with Lyme neuroborreliosis (early vs. late).

CSF parameter	Early neuroborreliosis (n = 37)	Late neuroborreliosis (n = 10)
Cell count/μL	218 (6–757)^a^	95 (23–312)^a^
Total proteins (g/L)	N/A	N/A
Albumin ratio (×10^−3^)	19.6 (8–58.4)^a^	45 (8–140)^a^
IgG synthesis rate	20% (17)^b^	50% (20)^b^
	81%	100%
IgM synthesis rate	54% (32)^b^	9% (13)^b^
	84%	40%
IgA synthesis rate	7% (17)^b^	39% (28)^b^
	19%	80%
Lactate	N/A	N/A

N/A, Not Available.

a Interval.

b Mean (±SD).

**Table 12:** Antibody detection and test sensitivity based on disease stage (adapted from Guntinas-Lichius & Schaitkin, 2016).

Stage	Immune response	Sensitivity/highlights
Early localized infection	After 3-weeks: IgM antibodies (not always detected, e.g., reinfections);	20% to 50%
	After 6-weeks: IgG antibodies (VIsE-IgG often detected early)	IgM predominance
	Some patients with brief symptoms may not have IgM or IgG	
Early disseminated infection	Similar to early localized infection	70% to 90%
	After 2-weeks of illness: production of intrathecal antibodies in cases of neuroborreliosis, >99% detectable after 6 weeks	Presence of IgM and IgG, predominance of IgG in longer lasting diseases
Late infection	High levels of IgG antibodies	Close to 100%
	Variable amount of IgM antibodies	Only IgG is relevant
	Intrathecal IgG antibody production with late Lyme borreliosis	

EMG, Electromyography.

**Figure 9:**
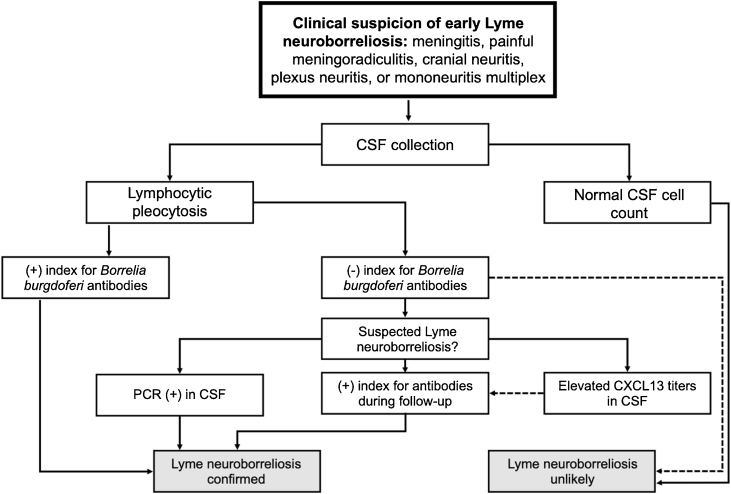
Flowchart for assessment of Lyme neuroborreliosis.

Regarding viral etiologies, PCR and serology for VZV, HSV, and enteroviruses can be used.^174^ In addition to Lyme disease, suspected meningitis and Guillain-Barré syndrome also warrant performing a lumbar puncture with CSF analysis.^4^ It is noteworthy that ordering laboratory tests, such as immunology, endocrinology, audiometry, and viral assessments, has not altered the initially prescribed treatment, which should not be delayed, as their usefulness lies in defining the etiology (Fig. 9).^72^

### Treatment

The treatment of Lyme disease is still supported by little evidence on the pediatric population, with much of the knowledge extrapolated from the results of studies in the adult population.

In suspected cases without a confirmed diagnosis, but without evidence of another disease despite thorough differential diagnosis, antibiotic therapy should be considered.^172^ In cases of early neuroborreliosis, antibiotic therapy should last 14 days; in late cases, treatment duration can be extended up to 21 days, with the following options: doxycycline, children aged nine years and older, at a dose of 4 mg/kg/day, with a maximum dose of 200 mg/day; ceftriaxone 50 mg/kg/day; cefotaxime 100 mg/kg/day; and penicillin G 200–500,000 IU/day.^172^, ^173^, ^175^

Corticosteroid therapy is contraindicated in suspected Lyme disease, where treatment should be performed with antibiotic therapy.^4^, ^176^, ^177^

**Figure 10:**
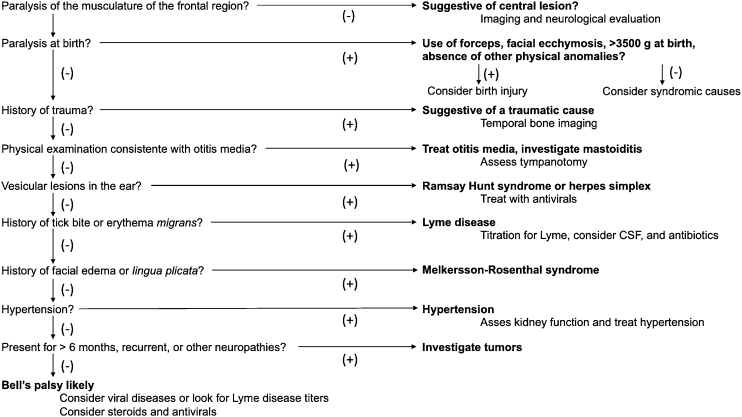
Algorithm for etiological diagnosis of peripheral facial paralysis in children.

In patients with acute otitis media, myringotomy with or without ventilation tube insertion is indicated, with subsequent culture-guided antibiotic therapy.^5^, ^178^ In patients with mastoiditis, mastoidectomy combined or not with myringotomy with or without tube insertion is indicated, with subsequent culture-guided antibiotic therapy (Fig. 10).^5^, ^178^

Regarding recovery, the pediatric population has higher recovery rates than the adult population, reaching up to 89.4% of complete recovery after two months. The higher recovery rate is attributed to the shorter length of the facial nerve in children and a greater capacity for axonal regeneration.^72^

### Recommendations

I – PFP in children differs in epidemiology and etiology from PFP in adults. The diagnosis should be based on the prevalence of local infection as well as abnormal findings in medical history and physical examination suggestive of specific pathologies, such as lymphoproliferative disorders, malignancies, and autoimmune diseases (Strong recommendation; High-quality evidence).

II – The treatment of facial palsy in children is directly related to the cause. Systemic or localized infections (such as mastoiditis) should receive specific antibiotic therapy and surgical treatment (Strong recommendation; High-quality evidence).

III – Myringotomy with or without tube insertion is an option, although a mastoidectomy and antrostomy is advised to be performed in cases of facial palsy in children (Weak recommendation; Moderate-quality evidence).

IV – Corticosteroid therapy is not indicated in cases of Lyme disease (antibiotic therapy is suggested instead) (Weak recommendation; Moderate-quality evidence).

## Evidence-based rehabilitation and treatment of peripheral facial palsy

### Surgical decompression of the facial nerve

Although Bell’s palsy is common, the absence of an established etiology means that treatment continues to be based on the presumed pathophysiology of edema and nerve entrapment in the Fallopian canal. Early treatment with prednisolone significantly increases the chances of complete recovery to 94% within the first nine months.^81^, ^84^ Reviews on the use of corticosteroids^82^ and antivirals^90^ are consistent with these findings. However, because the pathophysiology involves nerve entrapment, some surgeons have suggested that surgical decompression of the facial nerve is a suitable treatment option. There is no consensus on surgical decompression of the facial nerve in Bell's palsy. For patients at risk of poor recovery, surgical decompression of the facial nerve has been proposed as an additional treatment option to release the nerve from the Fallopian canal and improve outcomes.^179^, ^180^, ^181^, ^182^

Surgical decompression of the facial nerve for Bell’s palsy was first described in 1932. Ballance & Duel^183^ recommended cutting the sheath in the distal segment of the mastoid portion of the facial nerve, which was consistent with theories of injury site at the time. In the following decades, the proposed site for the operation migrated from 1 cm distal to the stylomastoid foramen to 1 cm distal to the Fallopian canal.^183^, ^184^ The diameter of the facial nerve is narrowest (approximately 0.69 mm) at the labyrinthine segment, where it enters the Fallopian canal, and inflammation and edema are more likely to occur at this point. In addition, a narrower part of the arachnoid is located at the proximal extension of the labyrinthine segment of the Fallopian canal and acts as a point of nerve compression.^185^ Thus, edema in the labyrinthine segment can lead to a reduction in axoplasmic flow due to pressure on the Fallopian canal, which can cause interruption of vascular flow, ischemia, and Wallerian degeneration distal to the compression point.^78^ In 1972, Fisch & Esslen^186^ demonstrated intraoperative changes in evoked EMG and edema proximally to the geniculate ganglion in 94% of evaluated patients. These findings were supported by gadolinium-enhanced MRI studies showing nerve enhancement in the distal IAC and labyrinthine/geniculate segments, which correlate with the areas of greater inflammation and edema.^187^ Enhancement was reported in these segments in cases of both acute (less than four weeks of onset) and long-term (more than four weeks of onset) Bell’s palsy, with gadolinium enhancement noted in all late cases up to four months after onset.^187^ In addition, Yanagihara et al.^179^ observed facial nerve edema in patients operated within three months of paralysis onset.

Based on these findings, it would be logical to decompress the facial nerve at the most affected segments. Although there are multiple approaches, Fisch & Esslen^186^ and May & Hawkins^188^ were the first to describe the two most common surgical approaches: the transmastoid and the Middle Fossa (MF) craniotomy. Transmastoid decompression provides better access to the tympanic and mastoid segments of the facial nerve.^179^ However, because incus injury or disarticulation is a possibility, conductive or sensorineural hearing loss is a common postoperative complication.^189^ In 1984, May et al.^190^ suggested that the transmastoid approach was beneficial for decompressing the labyrinthine segment. Conversely, MF craniotomy provides exposure of the labyrinthine segment, the geniculate ganglion, and the tympanic segment.^191^ However, the anatomical landmarks are more challenging to locate, and the procedure is quite invasive as it may require temporal lobe retraction.^191^ The occurrence of iatrogenic facial nerve injury, hearing loss, and/or vestibular dysfunction is rare when the procedure is performed by an experienced surgeon. Other risks associated with MF craniotomy such as CSF leak, meningitis, and complications associated with temporal lobe retraction (e.g., aphasia, seizure) are also rare but equally serious.^182^, ^192^ There are only a few prospective studies evaluating the effectiveness of decompression by MF craniotomy.^9^, ^180^ In a meta-analysis of very heterogeneous studies, Lee et al.^193^ demonstrated that facial nerve decompression by a combined transmastoid and MF approach 14 days after complete facial palsy onset is superior to clinical treatment. Given the significant risks and complications associated with both procedures and the lack of evidence demonstrating efficacy, neither approach is routinely recommended.

Opinions about the optimal timing of surgery and surgical approach are divergent. Early studies from the 1930s advocated decompression within three months after paralysis onset.^194^ Some authors argue that the most favorable results are obtained when decompression is performed within 14 days of onset,^180^, ^195^ while others suggest that delayed surgery is more beneficial (between three weeks and four months after onset).^179^, ^196^ Nonrandomized studies report conflicting results regarding the optimal timing of surgery. Giancarlo et al.^197^ and Yanagihara et al.^179^ reported improved recovery in a percentage of patients operated on within three months of onset. Another study advocates surgical decompression within 24 h of neural degeneration reaching 90%–94% within one to 21 days after paralysis onset.^9^ However, because nonsurgical treatment of Bell’s palsy is associated with favorable outcomes, the uncertainty regarding the affected segment of the facial nerve, optimal timing of surgery, and potential injury to the facial nerve and other ear structures during the surgery raises continuous discussions about whether surgery has a role in the management of this condition.^198^, ^199^

The use of electrophysiological tests in patients with severe Bell’s palsy may help identify those most likely to have a poor prognosis.^81^, ^200^ ENoG is the most popular technique used to evaluate patients with PFP.^201^, ^202^ When there is 95% nerve degeneration on ENoG, the patient has a 50% chance of a poor outcome (less than 50% chance of recovery to HB grade I or II) and would potentially benefit from surgical intervention.^180^, ^186^, ^201^, ^203^ Voluntary EMG measures motor activity with needle electrodes placed on the *orbicularis oris* and *orbicularis oculi* muscles while the patient is asked to make vigorous facial contractions. In the acute phase of paralysis, EMG is used when ENoG shows neural degeneration equal to or greater than 90% to confirm the absence of muscle function.^204^ The presence of active motor units on EMG in the setting of severe degeneration on ENoG indicates a phenomenon called deblocking, which is the asynchronous discharge of regenerating nerve fibers that do not produce a measurable CMAP on ENoG.^204^ Deblocking is a sign of neural regeneration and portends a favorable prognosis. Therefore, patients with deblocking should not proceed with surgical decompression despite severe degeneration on ENoG.^182^

Despite debates over different surgical approaches and timing, evidence for surgical decompression of the facial nerve in Bell's palsy is scarce. Few large studies have been conducted. May et al.^205^ stated that surgery had no place in the management of Bell’s palsy. In a multicenter study, Gantz et al.^180^ evaluated 30 patients treated for Bell’s palsy for 15 years. Inclusion criteria were onset of paralysis within 14 days, complete paralysis (HB grade VI), degeneration greater than 90% on ENoG, and absence of voluntary activity on EMG. Patients who met the inclusion criteria could choose between surgery (n = 19) and treatment with oral corticosteroids (n = 11). Another group of patients (n = 7) underwent surgery 14–28 days after paralysis onset. The surgical intervention consisted of facial nerve decompression by MF craniotomy, including decompression of the meatal foramen, labyrinthine segment, geniculate ganglion, tympanic segment, and distal portion of the IAC. In the nonsurgical control group (corticosteroids alone), no patients had improved to HB grade I, 4 of 11 (36%) had improved to HB grade II, and 7 of 11 (64%) had improved to HB grade III at 7-month follow-up. In the cohort that underwent decompression after 14 days, two patients had a good outcome (HB grade I) and five patients had a poor outcome. Of the patients who underwent decompression within 14 days, 18 of 19 (94%) had a good outcome and one patient had a poor outcome (HB grade III). The benefit of surgical decompression was statistically significant (*p* = 0.0001). Similar results were found in other studies.^9^, ^197^ Conversely, some studies found no differences between the surgical and control groups.^190^, ^206^, ^207^ It should be noted that, in these observational studies, both the electrophysiological assessment of nerve function at the time of surgery in relation to the onset of paralysis and which segments of the facial nerve were decompressed varied due to different surgical approaches.

The few randomized studies available included a small number of participants and did not have sufficient statistical power to detect the magnitude of the effect that could be expected, in addition to other limitations such as the choice of randomization method, follow-up time, and methodology for assessing the severity of facial palsy.^208^, ^209^, ^210^, ^211^, ^212^ The studies by Li et al.^209^ and Mechelse et al.^210^ used surgical approaches that did not decompress the affected segments of the facial nerve, which are considered the site of injury in Bell’s palsy. It is difficult to draw conclusions about surgical decompression based on available data, as other surgical approaches such as MF craniotomy, which typically provides better access to the affected segments, carry a higher risk of complications due to their aggressiveness compared with the retroauricular approach described in both studies. Furthermore, this side effect alone makes it difficult to justify surgical decompression if the patient has not fully recovered yet.^210^

### Recommendations

I – Surgical decompression is not a first-line treatment for Bell’s palsy (Strong recommendation; Moderate-quality evidence).

II – There is not enough evidence available about the indications of surgical intervention in Bell’s palsy. Current evidence is controversy to make recommendations for clinical practice (Insufficient evidence).

III –The existing heterogeneity in the current literature regarding recovery, classification scales, and schedules for measuring outcomes hinders the elaboration of recommendations about optimal timing of surgical decompression and surgical approach.

## Facial reinnervation by nerve grafting

The cause of irreversible facial nerve palsy can be surgical, traumatic, or idiopathic. For example, in surgical interventions, the resection of tumors that are close to the facial nerve, especially vestibular schwannomas, may result in neural damage.^213^

Repairs and nerve grafts are among the techniques that act directly on the facial nerve. They seek to restore facial movement and are considered dynamic techniques of facial rehabilitation. Hypoglossal-Facial Anastomosis (HFA) is one of the main treatments for the rehabilitation of patients for whom end-to-end anastomosis of the facial nerve is unfeasible. Other options such as Cross-Facial Nerve Grafting (CFNG) can also be used for dynamic facial rehabilitation.^214^

### Hypoglossal-facial anastomosis

The use of the hypoglossal nerve for facial nerve reinnervation was first described by Körte,^215^ in 1903, and several techniques have been described since then. HFA may be indicated in the presence of PFP with an unfavorable prognosis after approximately six months to one year of evolution, especially when the facial musculature still has a motor end plate capable of being innervated. It can also be indicated in cases where the possibility of end-to-end anastomosis is technically unfeasible. In these circumstances, the hypoglossal-facial anatomosis can be performed at the same time of surgical decompression to minimize the time that the patient will remain with the face paralyzed.

There are several surgical techniques for HFA. Sood et al.^216^ demonstrated good results on the HB scale, but with mobility disorders and tongue atrophy. Although they describe different intervals between VS resection and HFA, the main studies report better outcomes for patients who underwent HFA within two years of vestibular schwannoma removal.^217^ This period was previously proposed by Conley and Baker,^218^ in an analysis of 137 patients, of whom 16 developed facial palsy after vestibular schwannoma resection and were treated with HFA. In addition, it was also demonstrated that a shorter interval between vestibular schwannoma resection and HFA leads to better functional results.

Arai et al.^219^ investigated the use of split HFA in the rehabilitation of facial palsy after vestibular schwannoma resection. The results showed that all eight patients evolved from HB grade V or VI to grade III after the intervention, with mild or moderate atrophy of half of the tongue that resolved within one year. Mass movements associated with eating and talking were observed in two cases, but neither patient complained. The procedures were performed within six months after vestibular schwannoma resection, corroborating other findings that favor better outcomes in recently injured nerves.^220^

Zhang et al.^221^ presented a new surgical rehabilitation technique for facial nerve palsy after vestibular schwannoma surgery consisting of anastomosis of a pre-degenerated nerve graft proximal to the hypoglossal nerve and distal to the injured facial nerve. Ten out of 12 participants in the intervention group were rehabilitated after the follow-up period, and five were HB grade II. The authors attribute the lack of success in the rehabilitation of the other two patients to the duration of paralysis longer than one year. No participant had any side effect related to hypoglossal nerve injury.

Han et al.^222^ compared the outcomes of three HFA techniques performed after vestibular schwannoma resection. The case series evaluated 14 patients, of whom seven underwent the end-to-end technique, three underwent the end-to-side technique, and four patients underwent the split technique. All patients achieved good facial outcomes regardless of the technique used. Regarding tongue morbidity, more than half of the patients achieved satisfactory results. However, when compared with other techniques, patients who underwent end-to-side anastomosis had 100% favorable outcomes, whereas those undergoing the split and end-to-end techniques had 75% and 28% favorable outcomes, respectively. There was no significant difference between facial and tongue outcomes between the techniques, as well as tumor size and interval between vestibular schwannoma resection and HFA. Despite this, the authors selected the split technique as the most convenient, when possible, as it is associated with good results in facial mobility and tongue trophism. Regarding the interval between procedures, better outcomes were obtained in patients who underwent HFA up to 12 months after vestibular schwannoma resection, as other studies have already suggested.^220^

González-Darder et al.^213^ evaluated the efficacy of end-to-side HFA for facial nerve reanimation in 16 patients with irreversible facial palsy due to vestibular schwannoma resection. All patients showed improvement in facial movements (15 to HB grade III and one to HB grade IV). There were no cases of tongue atrophy and synkinesis. Mean time between vestibular schwannoma resection and anastomosis was approximately 8 months, with an interval of 3–16 months. Dabiri et al.^223^ analyzed 10 cases of facial palsy, of which two were secondary to vestibular schwannoma resection. Patients had a good facial mimicry response (HB grade III), with no speech or swallowing complications secondary to tongue atrophy. These findings are in accordance with other studies in the literature, demonstrating that HFA is a feasible option for improving paralysis and reducing morbidities.^224^

Hammerschlag ^225^ compared end-to-end HFA vs. Jump Interpositional Graft Hypoglossal Facial Anastomosis (JIGHFA) in the rehabilitation of facial palsy after surgery. Eighteen patients underwent JIGHFA, of whom 13 developed facial palsy after vestibular schwannoma resection. The comparison group consisted of 30 patients from a series by Brudny ^226^ and 22 patients who responded to an anonymous questionnaire about their perception after rehabilitation surgery by end-to-end HFA associated with EMG. Patients undergoing JIGHFA and those from the Brudny series achieved HB grades ranging from II to IV. Of patients who were subjectively assessed, 15 had good results (HB grade II–IV) and seven had undesirable results (HB grade V). Regarding morbidities, ophthalmic complaints were more prevalent among the classic HFA group (with complaints such as drooping eyebrows and eyelids, synkinesis when closing the eyes, visual impairment, and chronic tearing). Of note, 17 patients in the JIGHFA group and in the classic HFA group underwent gold weight lid implantation on the affected side. Regarding hypertonia, there was a higher prevalence among patients undergoing classic HFA, while synkinesis was more prevalent in the JIGHFA group. Speech and swallowing complications related to tongue morbidity were not observed in the JIGHFA group but were reported by 10 of the 22 patients who underwent classic HFA.^225^, ^226^ The comparison of these findings with other published studies evaluating the use of grafts in HFA shows good results in the recovery of facial mimicry with few or no problems related to tongue atrophy, but with the presence of synkinesis.^227^

Pardo-Maza et al.^228^ evaluated a retrospective cohort of 50 patients who underwent different facial reanimation procedures. Only eight patients underwent HFA, of whom four patients underwent end-to-end and the other four underwent hemi end-to-end HFA. The results were not reported separately, which precludes any analysis of the efficacy of either technique. However, the authors provide a decision-making algorithm for HFA that considers factors such as nerve integrity verified by electrical stimulation, possibility of spontaneous recovery, and availability of segments of the sectioned nerve.

### Masseteric-facial anastomosis

The motor nerve of the masseter muscle is a branch of the mandibular division (V3) of the trigeminal nerve. It is being increasingly used for facial reanimation and is the preferred option by some authors because of minimal donor site morbidity and consistent anatomical location.^229^

In the technique described by Faria et al.,^230^ in 2010, the peripheral branches of the extraparotid facial nerve are identified and dissected retrogradely, and the zygomatic and buccal branches are sectioned. The masseteric branch is accessed through a preauricular incision with elevation of the skin flap over the parotid gland, followed by a transverse incision in the parotid capsule approximately 1 cm below the zygomatic arch and 3 cm anterior to the tragus. Blunt dissection of the masseter is then performed through the parotid tissue to avoid sectioning the branches of the facial nerve. After it is located, the masseter muscle is dissected to reach its deepest portion and a nerve stimulator is used to locate the masseteric branch. The facial nerve is typically located approximately 1.5 cm below the SMAS. Once the nerve is identified, it is followed anteriorly to its ramification. An unbranched segment of approximately 1 cm is usually found, and it can be sectioned distally and rotated laterally toward the distal stump of the facial nerve. End-to-end anastomosis is performed with epineural sutures of 10–0 Nylon, if possible interrupted. As with HFA, physiotherapy is essential in the rehabilitation process.^230^

Murphey et al.^231^ performed a systematic review and meta-analysis on masseteric nerve transfer. Thirteen articles were included, with a total of 183 patients undergoing masseteric nerve surgery. Mean duration of paralysis prior to the intervention was 14 months, and the most common cause of paralysis was cerebellopontine angle tumors. Six studies performed coaptation of the main facial nerve trunk to the masseteric nerve, whereas the remaining seven studies used distal branches (zygomatic/buccal). Mean facial nerve recovery time was 4.95 months in all studies. Participants were separated into groups, main vs zygomatic/buccal branch and use of interposition graft, and subgroup analyses were performed. The main branch group had a delayed nerve recovery time of 5.76 months compared with 3.76 months in the distal group. However, this finding was not statistically significant and a difference between the coaptation of the main nerve or the distal branch could not be inferred. The use of interposition graft significantly delayed time of nerve recovery (6.24 months for the main vs. 4.06 months for the distal group), and this finding was statistically significant. This corroborates the findings previously described regarding the use of interposition grafts in direct facial nerve repair.^231^ Comparisons between other types of nerve transfer are rare, but Albathi et al.^232^ reported a faster recovery time for masseteric compared with hypoglossal nerve transfer (5.6 vs. 10.8 months, respectively).

Another benefit of masseteric nerve transfer is relatively low morbidity. Murphey et al.^231^ described only 12 complications reported in 183 patients, including masseter atrophy, ocular discomfort with chewing, surgical site infections, hematoma, sialocele, and otitis externa. Compared with hypoglossal transfer, the risk for donor morbidity is low because the nerve is sectioned distal to major motor components. Older patients who do not tolerate anesthesia may undergo the procedure under local anesthesia with light sedation.^233^ Finally, the rate of synkinesis is lower in masseteric nerve transfer.^234^

The main systematic review to compare HFA vs. Masseteric-Facial Anastomosis (MFA) was published in 2022 by Urban et al.^235^ The authors conducted a systematic review and meta-analysis to compare functional outcomes and adverse effects between the procedures. A review of online databases was performed to include studies with four or more patients undergoing hypoglossal or masseter nerve transfer without muscle transfer or other cranial nerve transposition. Facial nerve outcomes, time to reinnervation, and adverse events were pooled and studied. A total of 71 studies were included, of which 15 studies included 220 masseteric-facial transfers and 60 studies included 1,312 hypoglossal-facial transfers. Oral commissure symmetry at rest was better for hypoglossal transfer (2.22 ± 1.6 mm vs. 3.62 ± 2.7 mm, *p* = 0.047). The composite Sunnybrook Facial Nerve Grading Scale was better for masseteric transfer (47.7 ± 7.4 vs. 33.0 ± 6.4, *p* < 0.001). Time to first movement (in months) was significantly faster in masseteric transfer (4.6 ± 2.6 vs. 6.3 ± 1.3, *p* < 0.001). Adverse effects were rare (<5%) for both procedures. Therefore, both nerve transfer techniques are effective for facial reanimation, but the masseteric one has a faster recovery time.

### Contralateral neural graft (cross-facial nerve grafting)

Using the facial nerve from the normal hemiface may be an alternative in the treatment of facial palsy when the proximal stump of the facial nerve is not available for primary repair or when the proximal portion of the nerve presents irreversible degeneration. A sural nerve graft can be interposed between branches of the facial nerve from the normal hemiface and branches of the facial nerve from the paralyzed hemiface. This technique was described in 1975, by Scaramella,^236^ but is not commonly used, despite serving as an alternative to conventional techniques, especially in cases with less than one year of evolution in which the oral deformity is more pronounced.

In this technique, branches of the contralateral facial nerve are exposed through a standard preauricular incision. Stimulation of the nerve branches allows identifying which ones can be sacrificed without incurring functional damage to the normal side. In general, one or two branches that promote elevation of the labial commissure and upper lip are selected. The sectioned branches are then sutured end-to-end to a long sural nerve graft. Next, a subcutaneous tunnel is created in the upper lip, through which the graft is passed over to the paralyzed side of the face, where it will be anastomosed to the larger branch of the facial nerve.^237^ This technique can also be used as an initial preparation for the use of microvascular free nerve-muscle grafts in sequelae from long-term PFP.

Van Veen et al.^238^ compared patients who underwent HFA with those who underwent CFNG. Although no statistically significant differences were found, both techniques improve quality of life scores. An advantage of this approach is that patients can develop spontaneous and emotional movements.^239^ However, results have been varied. Axons can take up to nine months to cross long interposition grafts, and only up to 50% reach the distal nervous branches.^234^ Other pitfalls include weakening of the unaffected side and lack of energy, making it a less attractive option for the surgeon.^240^ Recent data on CFNG are lacking because there is greater interest in the HFA and MFA techniques.

### Recommendations

I – HFA and MFA can improve the degree of paralysis in most patients with long-term facial palsy (Strong recommendation; Low-quality evidence).

II – The earlier the procedure is performed, the better the functional recovery outcomes (Strong recommendation; Low-quality evidence).

III – MFA has a faster recovery time than HFA (Weak recommendation; Low-quality evidence).

IV –There is little published evidence on the CFNG technique, only from small case series (Insufficient evidence).

## Surgical treatment of long-term facial palsy

In general, facial palsy rehabilitation involves restoring neural control of paralyzed musculature or muscle transfers with the goal of achieving symmetry at rest and restoring movement dynamics. In cases of long-term facial palsy, only the second option is possible. Long-term facial palsy is usually defined as paralysis lasting over two years. Muscle groups that do not receive nerve stimuli for a prolonged period eventually become atrophic and cannot be stimulated again by surgical reinnervation. Another challenge is ensuring that the procedure performed can restore, in an aesthetically acceptable way, the symmetry and bilateral coordination of the face, which involves numerous muscle pairs. Therefore, the most complete restoration is achieved through muscle transfers, typically using microsurgical flaps. These flaps provide the best results regarding dynamic symmetry of facial movement. The first report of muscle transfer for rehabilitation of facial palsy was described by the New Zealander Harold Gilles, in 1934.^241^ The main indications for muscle transfer are absence of musculature (due to congenital or postablative causes) or muscle myopathies, in addition to prolonged periods of denervation.^242^

The different nervous origins in muscle transfers lead to fundamentally different rehabilitation results. When the transferred muscle is innervated by branches of the contralateral facial nerve, facial expressions show a certain degree of synchrony between both sides of the face, unlike with other reconstructions, such as those involving the masseteric nerve, where mastication muscles are involved. Thus, in these cases, the transferred muscle will be innervated by branches of the contralateral facial nerve or from branches of the trigeminal nerve, such as the masseteric nerve.

In addition to muscle transfers and transpositions, there are several other surgical procedures for the treatment of facial palsy. The goal of these procedures is to reestablish tonus and passive symmetry, in addition to the movement itself. They are typically divided into dynamic and static.

### Dynamic procedures

#### Microsurgical flaps

Several muscles can be transplanted for reanimation in facial palsy: the *latissimus dorsi*, *pectoralis minor*, *rectus abdominis*, among others. However, the *gracilis* muscle is one of the most used options for facial palsy rehabilitation.^243^, ^244^, ^245^ Its use was first reported by Harii et al.,^246^ in 1976.

The *gracilis* muscle is located on the medial compartment of the thigh, posterior to the *adductor longus*. It originates at the pubic symphysis and inserts into the medial condyle of the tibia. It is heavily supplied by several arterial pedicles in deep regions of the muscle. Its main pedicle comes from the deep artery of the thigh, mainly via the branch to the adductors, and from the medial circumflex artery of the thigh, with two concomitant veins. It is innervated by a branch of the obturator nerve, and its tibial tendon has an important and close relationship with the saphenous nerve.^242^, ^247^ The *gracilis* muscle flap does not cause morbidity or functional damage to the donor site.^248^

In cases of Cross-Facial Nerve Grafting (CFNG), the sural nerve is typically used for interposition grafting. The sural nerve is extensive, and its removal has minor repercussions on the donor site.^242^ This nerve graft is used for coaptation of the selected branches of the contralateral facial nerve to the *gracilis* muscle graft through the deep plane of the face, across the upper lip.^242^ The procedure is often performed in two stages. The first stage consists of obtaining the sural nerve graft, which is then inserted into a subcutaneous tunnel on the upper lip – its distal portion is sutured to the buccal branch of the facial nerve on the normal side while its distal end is anastomosed to the buccal branch on the paralyzed side. The second stage is performed between 4 and 12 months after the first stage, when axonal regeneration along the transplanted nerve is expected to have already occurred – the *gracilis* muscle is transferred, and microsurgical neurovascular anastomosis is performed. Blood supply and drainage are achieved through anastomosis involving the facial vein and artery.^243^ In addition, muscle volume should be reduced to adequately fit the face. Liang et al.^243^ performed two-stage CFNG in 12 patients and reported that all grafts remained viable, with excellent buccal movements in eight cases, good movements in three cases, and moderate movements in one patient. The static and dynamic angles of the lips were also improved.

Another advantage of CFNG reconstruction is related to movement spontaneity, which is an important factor in perceiving a smile as close to normal as possible. This spontaneity would be more difficult to achieve with other types of reconstructions.^249^

#### Muscle flap in the masseteric nerve

In cases of long-term palsy, the masseteric nerve, a branch of the trigeminal nerve, may also be used to innervate the muscle flap, notably the *gracilis* muscle. The masseteric nerve is located 3 cm anterior to the tragus and 1 cm inferior to the zygomatic arch, 1–2 cm below the muscular aponeurotic system in the masseteric muscle. This location is usually reproducible.^250^ The muscle is also innervated by a deeper nerve branch, located below the zygomatic arch, and preserved to maintain residual innervation.^250^ Once located, the nerve is dissected along its longest axis and sectioned to be anastomosed to the muscle flap.

There are clear distinctions between nerve reconstruction using CFNG and axon reconstructions in the masseteric nerve. Using the masseteric nerve precludes bilateral conjugate symmetry and requires activation by biting. However, spontaneity was observed in several cases, probably related to a phenomenon of neuroplasticity not fully elucidated. The benefits of using the masseteric nerve include a single-stage procedure and the preservation of the contralateral facial nerve.^251^ Use of the CFNG does not require neuroplasticity to produce conjugate movements but may sometimes produce different movement intensities and muscle activation, compromising symmetry. Muscle transfer is usually combined with midface lifting procedures to improve aesthetic outcomes, reestablish a nasolabial fold, and open the nasal airway.^242^

A very effective and objective method to assess recovery of facial function is to evaluate the extent of oral commissure movement. Variations in oral commissure position are measured at rest and with full smile. A group of investigators evaluated this parameter by comparing 70 procedures involving CFNG vs. 94 procedures involving the masseteric nerve in children.^249^ *Gracilis* muscle transfer was performed in all cases. Use of the masseter yielded better oral commissure movement outcomes compared with CFNG. Furthermore, the extent of oral commissure movement in the masseter group was similar to that of the normal side in the CFNG group, as shown by a commissure excursion in the range of normal.^249^ The *gracilis* muscle flap seems to yield a better and stronger response on the side innervated by the masseteric nerve. This would be explained by the following factors: (I) CFNG requires a two-stage neural reconstruction procedure, whereas the masseteric nerve graft only requires a one-stage procedure; (II) Nerve impulses need to cross longer distances when using CFNG compared with the masseteric nerve graft; and (III) The masseter muscle is more powerful than the muscles of facial expression, meaning that it could transmit stronger electrical impulses than the facial nerve.^249^ This very significant electrical impulse may result from the abundant presence of myelinated motor fibers (more than 2,000 according to a histological study).^250^

#### Temporalis muscle transposition

Rehabilitation of facial palsy using the temporal muscle usually uses the entire or part of the temporal muscle. The *temporalis* muscle is supplied by the deep temporal branch of the internal maxillary artery and innervated by the trigeminal nerve, which makes it more difficult to achieve movement spontaneity in reconstructions.^252^ *Temporalis* muscle transposition was first described by Gilles, in 1934, and was initially used for treating lagophthalmos.^241^ When biting, the muscle contracts and pulls the oral commissure up in a smile-like manner, just like it assists in the closure of the eyes when connected to the eyelid region. In these cases, a fascia lata graft is attached to the nasolabial fold.^241^ In this classic technique, the muscle is positioned over the zygomatic arch, which alone causes facial asymmetry. Several variations have been proposed since the early descriptions.^253^ In 1953, McLaughlin proposed orthodromic mobilization (that is, without inversion of the temporal muscle), in which the muscle flap is passed below the zygomatic arch and extended by a fascia lata graft transorally.^253^ More recently, Labbé and Huault^254^ described a new technique that consists of temporal muscle lengthening, in which the muscle tendon is sutured directly to the lips, without the need of a fascia lata graft. In this case, the zygomatic arch is sectioned.^254^ In addition, facial mimicry is not spontaneous, requiring physical rehabilitation, and a discreet scar is formed over the nasolabial fold.^255^ Another technique that does not require removing bone of the zygomatic arch and has minimal muscle manipulation has been described, which can be performed under local anesthesia.^253^

A systematic review comparing the outcomes of Lengthening Temporalis Myoplasty (LTM) vs gracilis free flap transfer in cases of long-term facial palsy showed that patients who underwent LTM had a lesser extent of smiling, suggesting that gracilis free muscle transfer is a better option for the treatment of long-term facial palsy. However, LTM should be considered a suitable alternative because it is less extensive, does not require muscle harvesting and microvascular anastomosis, and results in very high rates of spontaneous smiling.^256^

### Static procedures

Static procedures are aimed at repositioning or aligning face segments without promoting dynamic adequacy. They are often used in combination with dynamic procedures as a way of adjusting symmetry or for protection, such as corneal protection. There are numerous procedures and techniques, and we will discuss the most common and currently used ones.

#### Eyes

One of the most impactful consequences of facial palsy is impaired eye closure, or lagophthalmos, as a direct result of paralysis of the orbicularis oculi muscle. Lagophthalmos is the anomalous opening of the palpebral fissure with difficulty or inability to close the eyelids completely. Its main consequence is impairment of the ocular surface, in addition to aesthetic issues.

It should be noted that there is no instrument or objective method for measuring spontaneous and voluntary eye closure that is adequate to measure and compare the outcomes of procedures regarding inability of closing the eyelids. In addition to the closure itself, other aspects involved in eyelid closure include closure amplitude, spontaneity, and quality of life.^257^

##### Upper eyelid

Upper eyelid retraction in facial palsy results from the unopposed action of the *levator palpebrae superioris* muscle, which is innervated by the superior branch of the oculomotor nerve rather than the facial nerve.^252^ The principles of upper eyelid treatment basically involve the use of weights or other methods that exert gravitational force on the eyelid.

The use of gold weights to restore eye closure was first described by Illig,^258^ in 1958. However, the use of weights for eyelid closure was first described in 1927 by Sheehan,^259^ using a stainless-steel mesh. Gold was established as the standard material for this purpose because of its high density and malleability, in addition to its wide availability and relatively affordable price for the required quantity. For effective corneal protection, lower eyelid procedures are usually required, such as tarsal strip, tarsal strip combined with lateral tarsorrhaphy, or isolated lateral tarsorrhaphy.^260^

The gold weight is inserted into a pocket between the orbicularis muscle and the tarsal plate of the upper eyelid. It must allow eyelid closure without causing ptosis. Typically, the implants are slightly curved to minimize their prominence below the skin during movement.

Treatment failure or unfavorable results are mostly related to inadequate implant weights (gold or other materials). The use of 1.2 g of gold per implant yielded good results and a low revision rate.^261^ The most significant complication would be the possibility of migration and extrusion. In a series of 28 procedures involving gold eyelid weight, 19 complications (68%) were reported: 2 infections (7%), 5 dislodgements (18%), and 12 extrusions (43%). To reduce these complications, the authors suggest using the smallest weight necessary, placing it deeply through a medial incision, and securing it to the tarsal plate with a suture.^261^

Other materials have also been used to make eyelid weights, such as platinum. Because platinum is denser than gold, smaller and more discreet implants can be manufactured.^262^ In a series of 100 patients who received platinum eyelid weights, with 102 weights implanted in total, there was a low rate of complications (5.9%): three extrusions, two capsule formations, and one case of astigmatism. All the extrusions involved patients who underwent irradiation to treat malignant tumors.^262^ Compared with platinum, the only advantage of gold may be the lower price.^263^ An alternative for the treatment of lagophthalmos with weights would be the use of eyelid springs. However, these procedures are rarely used because of the relatively high complications rate.^263^

##### Lower eyelid

Paralysis of the lower eyelid results in lower eyelid retraction, bulbar conjunctiva, and cornea exposure, lagophthalmos, and inadequate tear drainage with watery eye sensation and epiphora.^264^ Some procedures that may control these manifestations include lateral canthoplasty, lower eyelid shortening, lower eyelid suspension, graft reposition, and midface lift.^263^, ^264^ Subperiosteal midface lift is effective because it moves large amounts of tissue up, eliminating any tension along the edge of the eyelid, in addition to improving the shape and projection of the zygomatic region and cheek.^264^

##### Tarsorrhaphy

Tarsorrhaphy is rarely performed and is mostly indicated when other techniques of corneal protection have failed. It can be performed by several temporary or permanent techniques. Temporary tarsorrhaphy basically involves suturing the upper and lower eyelids together, whereas long-term or definitive procedures are done by de-epithelializing the eyelid margins and joining the upper and lower eyelids.^265^ Long-term management of lagophthalmos with tarsorrhaphy is currently not recommended because it may reduce the visual field and produces unsatisfactory aesthetic results.^265^

#### Nose

Nasal implications of facial palsy are mostly related to the inferomedial displacement of the alar base, which contributes to the narrowing and collapse of the nasal valve. The most common treatment options for nasal valve collapse are the use of grafts, such as spreader and batten grafts, and nasal valve suspension. The nasal valve suspension technique was described in 1996, by Paniello.^266^ The upper lateral cartilage is pulled superiorly and laterally by a suture fixed close to the inferior border of the orbit.^266^ Nasal valve suspension allows a superolateral pull of soft tissue of the pyriform aperture region, resulting in a permanent effect of nasal valve opening, unlike grafting techniques, which although have a good effect on the opening of the nasal valve itself, have little or no effect on adjacent tissue.^267^ Nasal valve suspension surgery is a relatively simple procedure that can be performed under local anesthesia, with a low rate of complications and excellent short-term results, although these initial results seem to reduce over time.^267^

#### Frontal region

The primary function of brow lifting is to treat forehead and brow ptosis. The most significant consequence of brow ptosis is drooping of the upper eyelid, which, in addition to obvious aesthetic impairment, may also reduce the visual field. In unilateral facial palsy, the lack of resting muscle tone is often exacerbated by hyperactivity of the contralateral facial muscles.^268^ In these cases, with the aim of improving brow symmetry, temporary chemical denervation can be performed using botulinum toxin.^269^ Brow ptosis can be treated by different surgical techniques, especially brow lifting. Brow lifting may be performed as an open or endoscopic procedure, with good results and adequate patient satisfaction being reported in a case series of patients treated with endoscopic brow lifting.^268^

#### Mouth

Some previously mentioned procedures, such as muscle transfers and nerve reconstructions, have effects on the oral commissure. However, there are interventions specifically targeting the mouth, such as suspension of the oral commissure through sling techniques. These techniques involve the use of autologous grafts, such as the fascia lata, or synthetic materials (heterologous), such as Gore-Tex. The use of heterologous grafts eliminates donor site morbidity associated with the traditional harvest of autologous grafts but has a higher rate of complications, such as sling laxity and some cases of local infection.^270^ The aim of these techniques is to restore mouth and face symmetry by lifting the paralyzed facial muscles to counteract the effects of gravity and contralateral muscles.^271^ Therefore, in addition to bringing benefits to the oral commissure, they also benefit the forehead and cheeks. One technique involves using two fascia lata slings to fixate the upper and lower lips to the zygomatic arch subcutaneously. This procedure was shown to clearly improve facial symmetry, in addition to being uncomplicated and having low morbidity.^271^

Finally, the aim of facial reanimation in long-term facial nerve palsy is to restore the symmetry, spontaneity, and natural aspect of the face at rest and during movement. Muscle transfer is currently the gold standard treatment for long-term facial palsy. In addition to muscle transfers and transpositions, which are the main dynamic techniques, there are several static techniques that are useful for restoring the symmetry and natural aspect of the face.

### Recommendations

I – Dynamic procedures involving microsurgical free flap transfer can be indicated for facial reanimation in patients with long-term facial palsy (Strong recommendation; Low-quality evidence).

II – Procedures involving masseter muscle flaps can be indicated for patients with long-term facial palsy (Strong recommendation; Moderate-quality evidence).

III – *Temporalis* muscle transposition may also be indicated in cases of long-term facial palsy (Strong recommendation; Moderate-quality evidence).

IV – Static procedures aimed at aligning face segments, such as the use of gold weights to correct eyelid retraction, can be recommended for patients (Strong recommendation; Low-quality evidence).

V – Tarsorrhaphy could be recommended as a temporary procedure (Weak recommendation; Low-quality evidence).

VI –Nose (for pyriform aperture enlargement), frontal (for reducing upper eyelid ptosis), and oral commissure surgery can be recommended for patients (Strong recommendation; Low-quality evidence).

## Non-surgical rehabilitation of the facial nerve

Facial palsy is debilitating for most affected people, with emotional, physical, social, and financial implications. Facial palsy can result from several different conditions, and it may be central or peripheral, congenital, or acquired.^272^ Bell’s palsy is the most common type of facial palsy, with an incidence of 30 cases per 100,000 people per year.^59^ Approximately 70% of people affected by Bell’s palsy spontaneously recover in a few weeks without undergoing any treatment.^59^ Conversely, 30% of patients may only recover partially and develop sequelae, such as contractures and synkinesis.^59^

Nonsurgical rehabilitation is a valuable mechanism of PFP management. It allows patients to successfully manage their symptoms and actively improve facial function, even in long-term cases.^273^ Orofacial myofunctional therapy involves muscle preparation with exercises, patient education about what facial alterations are being treated, and targeted corrective functional training.^274^ Therapeutic planning should be individualized and target the specific difficulties identified. During patient assessment for possible facial reanimation, it is important to determine the underlying cause of the paralysis, the exact extent of the lesion, the time of symptom onset, the viability of the facial musculature, the presence and status of the facial and other CNs, the patient’s general health status, and the patient’s expectations and goals regarding rehabilitation.^273^, ^275^

The care protocol should consist of an initial assessment (take patient history and perform clinical examination, determine the stage of paralysis and which muscle segments are affected) followed by pre-intervention documentation, therapeutic planning (frequency of treatment sessions with specific muscle and functional planning based on the initial assessment and the goals to be achieved), periodic reassessments with documentation, and discharge or assisted completion of the process.^274^, ^275^, ^276^ However, all therapeutic processes depend on patient interest and adhesion. During treatment sessions, the patient should be motivated, especially regarding any functional and aesthetic gain, even if minimal.^274^

Assessment of facial musculature at rest consists of comparing the symmetry of facial lines and reference points between the affected and normal sides.^275^ It is recommended assessing the following facial lines and reference points: forehead lines, eyebrow position, position of the lower eyelid in relation to the horizontal plane, position of the nasal philtrum in relation to the vertical plane, position of the ala of the nose in relation to the horizontal plane, nasolabial fold, position of the oral commissure in relation to the horizontal plane, and chin symmetry. Findings may vary according to the stage of paralysis.^275^

Assessment of facial dynamics allows analyzing the action of each muscle (or muscle group), comparing the amplitude of movement on the affected side with the normal side.^275^ The following should be evaluated: brow elevation (*frontalis* muscle), brow contraction (*corrugator supercilia* muscle), natural eye closure (lacrimal portion of the *orbicularis oculi* muscle), forced eyelid closure (*orbicularis oculi* muscle), nasal elevation (*procerus*, *levator anguli oris*, and *levator labii superioris* muscles), closed protrusion with closed lips (*orbicularis oris* muscle), closed retraction with closed lips (*risorius* muscle), open protrusion with open lips (*orbicularis oris*, *levator anguli oris*, and *levator labii superioris* muscles), open retraction with open lips (*zygomaticus* major and minor, *levator labii superioris*, and *depressor labii inferioris* muscles), lip pucker (*orbicularis oris* muscle), sucking (*buccinator* muscle), tongue movement, chewing, and speech.^275^

Orofacial myofunctional therapy programs should involve patient education about paralysis, muscle training, massages, relaxation, and a personalized home program.^273^ Most studies report improvement in facial movements or function with the application of several combined methods, and these results seem to persist with continued rehabilitation.^273^ However, rehabilitation programs are not standardized with regarding, type of therapy, and duration and frequency of intervention. This heterogeneity and the methodological limitations affect the strength of the evidence and prevent a reliable comparison between methods.^272^

The main facial physical therapy techniques include massage, facial exercises, electrical stimulation, acupuncture, neuromuscular training with biofeedback, mime therapy, and proprioceptive neuromuscular facilitation.^277^ Table 13 lists some myofunctional rehabilitation programs for patients with facial palsy, and the main ones are discussed below.

**Table 13:** The most common training programs applied in patients with facial palsy.

Type of therapy	Characteristics
Neuromuscular biofeedback training	Muscle activity is objectively recorded using surface EMG. Used for the treatment of synkinesis. Its effectiveness has been demonstrated in randomized controlled trials
Proprioceptive neuromuscular facilitation	Stimulates the development of complex patterns of coordinated muscle actions, the normalization of muscle tone, muscle stretching, and muscle strengthening
Mime therapy	Based on mime training for actors, focuses on symmetry of the face at rest and during movement. Effectiveness has been shown
Electrical stimulation	No standards have been defined and effectiveness has not been shown
Acupuncture	Effectiveness has not yet been shown

### Neuromuscular training with biofeedback

Neuromuscular training with biofeedback is also known as neuromuscular re-education. It aims to facilitate the restoration of intended facial movement patterns and eliminate undesired patterns of facial movement and expression.^278^ It is mainly used for the treatment of facial palsy (little or no movement) and involuntary movements (synkinesis). The patient activates a muscle or muscle group (contraction) while inactivating the muscle or muscle group responsible for the involuntary movement (relaxation).^278^

Myofunctional therapy is performed with the aid of some biofeedback, either a mirror or surface EMG. Because it is more accurate and objectively records muscle activity, surface EMG is more indicated. Stand-alone EMG systems or more complex computerized systems with software for data analysis and selection, video camera for visual feedback, and microphone for auditory feedback are used.^277^ A surface and a reference electrode are placed on the face. Visual, auditory, and electrical feedback from facial muscles during movement or at rest direct myofunctional therapies.^277^ By receiving feedback, the patient can regulate the use and strength of muscle contraction.^276^ In addition, it allows objective representation of the rehabilitation progress, even in the apparent absence of facial movement, and provides prognostic information about functional recovery from nerve lesions.^276^, ^279^

Several investigators have described improvements in facial movement because of neuromuscular re-education using surface EMG biofeedback or mirror feedback. Most studies used retrospective data. The few randomized controlled trials available demonstrate the effectiveness of neuromuscular re-education in the treatment of PFP.^280^, ^281^, ^282^, ^283^

Nakamura et al.^280^ evaluated the efficacy of facial rehabilitation using mirror biofeedback in the prevention of synkinesis. In a randomized controlled clinical trial, 27 patients were divided into two groups: 12 patients were treated with the rehabilitation method and 15 served as controls and did not receive any treatment. Treatment consisted of 30 min of daily training for 10 months. The degree of synkinesis was evaluated by computing the percent asymmetry of eye-opening width. The results indicated a significant lower degree of synkinesis in the training group than in the control group (*p* < 0.05).

Ross et al.^281^ conducted a prospective randomized controlled trial to evaluate the efficacy of EMG biofeedback vs mirror feedback as treatment strategies for patients with long-term facial palsy (18 months minimum). Both EMG and mirror feedback groups showed improvements in symmetry of voluntary movements (*p* < 0.03) and linear measurement of facial expression (*p* < 0.01) compared with controls. The authors concluded that neuromuscular training combined with either EMG or mirror feedback in association with a home rehabilitation program is an effective treatment for patients with facial palsy.

Manikandan^282^ conducted a randomized controlled trial comparing the effect of facial neuromuscular re-education (using a mirror for visual feedback) vs conventional therapeutic measures (electrical stimulation, gross facial exercises, and massage) in improving facial symmetry in patients with Bell’s palsy. Changes in Facial Grading Scale scores were statistically significant (*p* < 0.01) between both groups, especially regarding improvement in movement symmetry after three months of therapy. Facial neuromuscular re-education was more effective than conventional therapeutic measures in improving facial symmetry in patients with Bell’s palsy.

Pourmomeny et al.^283^ conducted a randomized controlled trial to evaluate the use of neuromuscular training with EMG biofeedback in the control or prevention of synkinesis in patients with PFP. The experimental group (n = 16) was treated with EMG biofeedback, whereas the control group (n = 13) received only common physiotherapy. All patients were treated for one year. Mean Facial Grading Scale score after treatment was significantly higher in the experimental group (*p* < 0.05) than in the control group. In the experimental group, patients without synkinesis were almost twice the number of those in the control group (31% vs. 16%), whereas patients with severe synkinesis were only one-third of those in the control group (13% vs. 45%). Using biofeedback therapy before synkinesis would help prevent it in patients with facial nerve injury.

### Proprioceptive neuromuscular facilitation

Proprioceptive neuromuscular facilitation is also known as Kabat’s rehabilitation. This method was developed by Herman Kabat and Margaret Knott between 1946 and 1951, respectively.^284^ It aims to promote functional gain through the inhibition, relaxation, and strengthening of muscle groups. This technique helps control muscle tone, balance and coordination of movements, muscle synergy, and motor learning through neuroplasticity.^284^

The principles of Kabat’s rehabilitation are the following: resistance (assists in muscle contraction by increasing motor control), propagation and reinforcement (propagation of stimulus response), manual contact (stimulates sensory receptors and controls movement execution), verbal command (helps the patient perform the appropriate movement), visualization (visual feedback of the desired movement), traction and approximation (stretching and muscle compression with facilitation of movement and stability), stretching (muscle stretching), movement synchronization (coordination of movements of muscle groups), and standardization of facilitation for movement performance.^284^

Barbara et al.^285^ conducted a randomized controlled trial to evaluate the efficacy of early proprioceptive neuromuscular rehabilitation (Kabat’s rehabilitation) in patients with Bell’s palsy. Patients were divided into three groups: (a) Patients undergoing early rehabilitation, (b) Patients not undergoing rehabilitation, and (c) Group B patients who did not improve after 15 days and then underwent rehabilitation. Patients who underwent proprioceptive neuromuscular facilitation showed improved clinical recovery after 15 days of treatment. The improvement in HB grade obtained in patients who underwent rehabilitation was significantly better than that in patients who did not undergo rehabilitation.

In a prospective, nonrandomized, controlled trial, Monini et al.^286^ compared functional outcomes in severe cases of Bell’s palsy (HB grade IV and V) treated by steroids alone or by steroids in combination with Kabat’s rehabilitation. Patients who underwent Kabat’s rehabilitation were 20 times more likely to improve HB by three grades or more (OR = 17.73, 95% CI 5.72–54.98, *p* < 0.001) than patients who did not receive physical treatment, in addition to having a faster recovery time. The authors concluded that steroid treatment appears to provide better and faster recovery in severe cases (HB grade IV and V) of Bell’s palsy when complemented with Kabat’s rehabilitation.

Studies comparing proprioceptive neuromuscular facilitation with other treatment modalities are scarce. The studies applied Kabat’s rehabilitation in the early stage of Bell’s palsy. Despite the first study being a randomized controlled trial, it has a short follow-up period of only 15 days, which is not enough time for patients to go through the flaccid stage and develop possible sequelae.^285^ As for the second study, although it was prospective and controlled, it was not randomized, which may have introduced selection bias.^286^

Despite the methodological limitations, facial rehabilitation using Kabat’s method shows promising results. The replication of these studies by well-designed, blind, randomized controlled trials could increase the evidence of the efficacy of this treatment in the early phase of PFP.

### Mime therapy

Mime therapy is a rehabilitation technique developed in the 1970s in the Netherlands by actor Jan Bronk and otorhinolaryngologist Pieter Devriese.^287^ The method focuses on restoring facial symmetry at rest and during movement through training of facial expressions and functional movements. Training includes facial massages; breathing and relaxation exercises; specific facial exercises; lip movement exercises for eating, drinking, and rinsing the mouth; speech exercises with pronunciation of letters and words; emotional expression; and guidance.^287^

Beurskens and Heymans^288^ evaluated the efficacy of mime therapy in a randomized controlled trial in the treatment of long-term PFP (paralysis lasting more than nine months). The experimental group received mime therapy consisting of massage, relaxation, inhibition of synkinesis, and coordination and emotional expression exercises. The control group did not receive any treatment. After three months of mime therapy, the experimental group had improved their facial symmetry (95% CI 10.4–30.4) and reduced the severity of their paresis (95% CI 0.1–1.1) compared with the control group. Mime therapy improved facial symmetry and reduced the severity of paresis in patients with facial nerve paresis. The protocol used by Beurskens and Heymans consisted of six steps: (1) Daily face and neck massage for 10−15 min followed by stretching of the affected side to relieve the muscles involved in synkinesis; (2) Recognition of muscle tensions (general and facial musculature) and breathing and relaxation exercises; (3) Exercises to co-ordinate both halves of the face and to decrease synkinesis with the use of mirror feedback; (4) Eye and lip closure exercises; (5) Exercises to increase awareness of lip movements and the position of the mouth for various sounds; and (6) Facial expression exercises.^288^

Mime therapy is effective for improving functional outcomes in patients suffering sequelae from PFP. It is inexpensive, widely available, and should probably be included in rehabilitation programs. The replication of this therapy in other groups could increase the strength of the evidence for the efficacy of this therapy in the treatment of PFP.

### Electrical stimulation

Electrical stimulation for the treatment of facial palsy has been used since the 1950s. There are several types of electrical stimulation, including galvanic, eutrophic, monophasic, biphasic, subthreshold, and contraction level.^289^ It aims to encourage axonal regeneration and promote the contraction of muscles that have lost innervation, reducing the recovery time from the injury, and strengthening the muscles during the acute phase of facial palsy.^289^ However, the use of electrical stimulation for PFP is controversial. For some authors, the use of nonspecific electrical stimulation in the peripheral facial neuromuscular system during the recovery process reinforces abnormal patterns (synkinetic) of facial muscle activity.^278^

Systematic reviews of randomized and quasi-randomized controlled trials revealed that electrical stimulation treatment guidelines lack standardization.^289^, ^290^, ^291^, ^292^ In addition, they do not demonstrate a significant advantage of using electrical stimulation in the treatment of facial palsy regardless of the stage. Several methodological problems were found, such as: use of inappropriate randomization methods, difficulty in blinding therapists and patients, small number of patients in some groups, variation in intervention protocols with different duration and frequency of treatment, and very short follow-up periods.^289^, ^290^, ^291^, ^292^

Mosforth and Taverner^292^ conducted a randomized controlled trial to evaluate the efficacy of electrical therapy in the treatment of Bell’s palsy. The controls (n = 40) were instructed to massage the face daily, while patients in the experimental group (n = 43) received electrical stimulation in addition to massage. The treatment period varied from two to six months. The results did not show an advantage of using galvanic electrical stimulation for the treatment of Bell’s palsy.

Alakram & Puckree^294^ investigated the safety and potential efficacy of using electrical stimulation in patients with Bell’s palsy of less than 30 days’ duration in a quasi-randomized controlled trial. Both groups were treated with heat, massage, facial exercises based on neuromuscular re-education training, and a home program. The experimental group also received electrical stimulation. The difference between the two groups was not statistically significant (*p* = 0.36). Electrical stimulation as used in the study during the acute phase of Bell’s palsy was safe but did not add value over spontaneous recovery and multimodal physiotherapy.

In a randomized controlled trial, Tuncay et al.^295^ investigated the efficacy of monophasic electrical stimulation over conventional physical therapy in patients with acute Bell’s palsy. All patients were treated with oral corticosteroids, beginning at a dose of 60 mg/day within the first 48 h after symptom onset. The first group received physical therapy applying hot pack, facial expression exercises, and massage to the facial muscles, whereas the second group received electrical stimulation treatment in addition to the physical therapy, five days per week for a period of three weeks. After treatment, the second group had a greater improvement in HB grades compared with the first group (*p* =  0.03).

A systematic review conducted by Fargher and Coulson^289^ with the objective of determining the effectiveness of electrical stimulation in the treatment of PFP included four acute phase studies.^289^, ^293^, ^294^, ^295^, ^296^ When the study data were pooled for meta-analysis, there was no statistically significant difference between the treatment and control groups. There is not sufficient evidence to support the use of electrical stimulation in the treatment of Bell’s palsy or other causes of facial palsy.

### Botulinum toxin

Botulinum toxin is obtained from *Clostridium botulinum* and consists of a pharmacological treatment that promotes temporary chemical denervation in the muscles.^275^ Functionally, botulinum toxin acts on the neuromuscular junction by blocking the presynaptic release of acetylcholine (the neurotransmitter responsible for promoting muscle contraction), generating temporary paralysis on the injected area.^275^, ^277^, ^297^

The effect of botulinum toxin on the musculature is progressive and starts to take effect in three to five days. The peak effect is routinely seen at two weeks.^277^, ^297^ Depending on the efficacy, the dose can be corrected within 14 days.^275^ Clinically, it takes approximately six to eight weeks for muscle action to become significant again. When symptoms resume, a new application can be performed to maintain the therapeutic effect.^275^

There are two serotypes commercially available for clinical use: type A and B.^275^ Botulinum toxin type A is the most widely used and is approved in most countries. Type B has lower potency and is an option in cases of resistance to botulinum toxin type A.^275^ The development of resistance or immunogenicity of botulinum toxin is uncommon and is directly related to the amount and repetition of injections.^275^, ^277^ Thus, the patient should not receive a new injection before the effect of the last injection has worn off. Facial injection intervals of less than three months should be avoided.^277^

Botulinum toxin is very effective in the treatment of sequelae from facial palsy. The sequelae should be well diagnosed, both at rest and during movement. The patient’s face must always be recorded, as well as the doses for future applications.^275^, ^297^ In the presence of spasms or contraction, the main objective is to relax the musculature, which is achieved with an ipsilateral injection. In cases of facial asymmetry, the aim is to maintain facial movement as harmonic as possible, for which the application of botulinum toxin on the contralateral side is also effective.^275^

Randomized controlled trials demonstrated the effectiveness of botulinum toxin in the treatment of sequelae from PFP.^277^, ^297^, ^298^, ^299^ Borodic et al.^298^ conducted a double-blind randomized controlled trial with 36 patients to test the efficacy of botulinum toxin in the treatment of synkinesis. The study demonstrated a reduction in synkinesis and an improvement in quality of life, increasing the evidence for botulinum toxin application in the treatment of synkinetic movements in long-term facial palsy.

Pourmameny et al.^299^ evaluated the efficacy of botulinum toxin type A and neuromuscular training in the treatment of synkinesis in patients with more than one year of facial palsy in a randomized controlled trial. Botulinum toxin type A was injected into the synkinetic muscle in the experimental group, and patients in the second group also participated in neuromuscular retraining with EMG biofeedback. There was a greater reduction in synkinesis in the group treated with neuromuscular retraining combined with botulinum toxin application (*p* = 0.041) after four months of treatment.

Botulinum toxin has become the standard treatment for synkinesis, as it is relatively easy, requires no patient downtime, is quite reproducible, and has a low rate of risks and complications.^297^ This treatment can be optimized with the combined use of neuromuscular training with biofeedback.^277^, ^297^, ^299^

In summary, there is a paucity of well-designed studies in the medical literature that prove the efficacy of physical rehabilitation for facial palsy. Most studies have a retrospective design or report on case series. However, the few available randomized controlled trials have reported that some therapies are promising and effective in the treatment of facial palsy. The application of botulinum toxin, neuromuscular training, proprioceptive neuromuscular facilitation, mime therapy with surface EMG and/or mirror feedback, or supervised home exercise programs has positive results in the treatment of PFP, with improvement of asymmetry and reduction of synkinesis.

### Recommendations

I – Neuromuscular training with biofeedback has been shown to improve facial palsy (Strong recommendation; Moderate-quality evidence).

II – Proprioceptive neuromuscular facilitation and mime therapy are possible options for nonsurgical treatment of PFP (Moderate recommendation; Low-quality evidence).

III – Electrical stimulation is contraindicated in the treatment of PFP (Strong recommendation; Low-quality evidence).

IV – The use of botulinum toxin can be included in the treatment of facial palsy to reduce facial asymmetry (Moderate recommendation; Low-quality evidence).

## Conclusion

Although the etiology of PFP is diverse, it is often associated with neural edema within a bony compartment that reduces blood flow and consequently leads to Wallerian degeneration. There is more evidence supporting the use of drug treatments with systemic corticosteroids than surgical treatment. It is important to correctly identify the problem and start treatment early to achieve better results. Individualized treatment aimed at correcting long-term sequelae is recommended and should be discussed with those patients with unsatisfactory recovery from facial palsy.

## Funding

The authors have no financial relationships relevant to this article to disclose.

## Conflicts of interest

The authors declare no conflicts of interest.
