# Supplementary material for: Task force of the Brazilian Society of Otology — evaluation and management of peripheral facial palsy
Source: Braz J Otorhinolaryngol. 2023 Dec 8;90(3):101374. doi: 10.1016/j.bjorl.2023.101374 (PMC10884764; doi:10.1016/j.bjorl.2023.101374)

BJORL-D-23-00280_Supplementary Material

**Appendix 1** Sunnybrook scale.


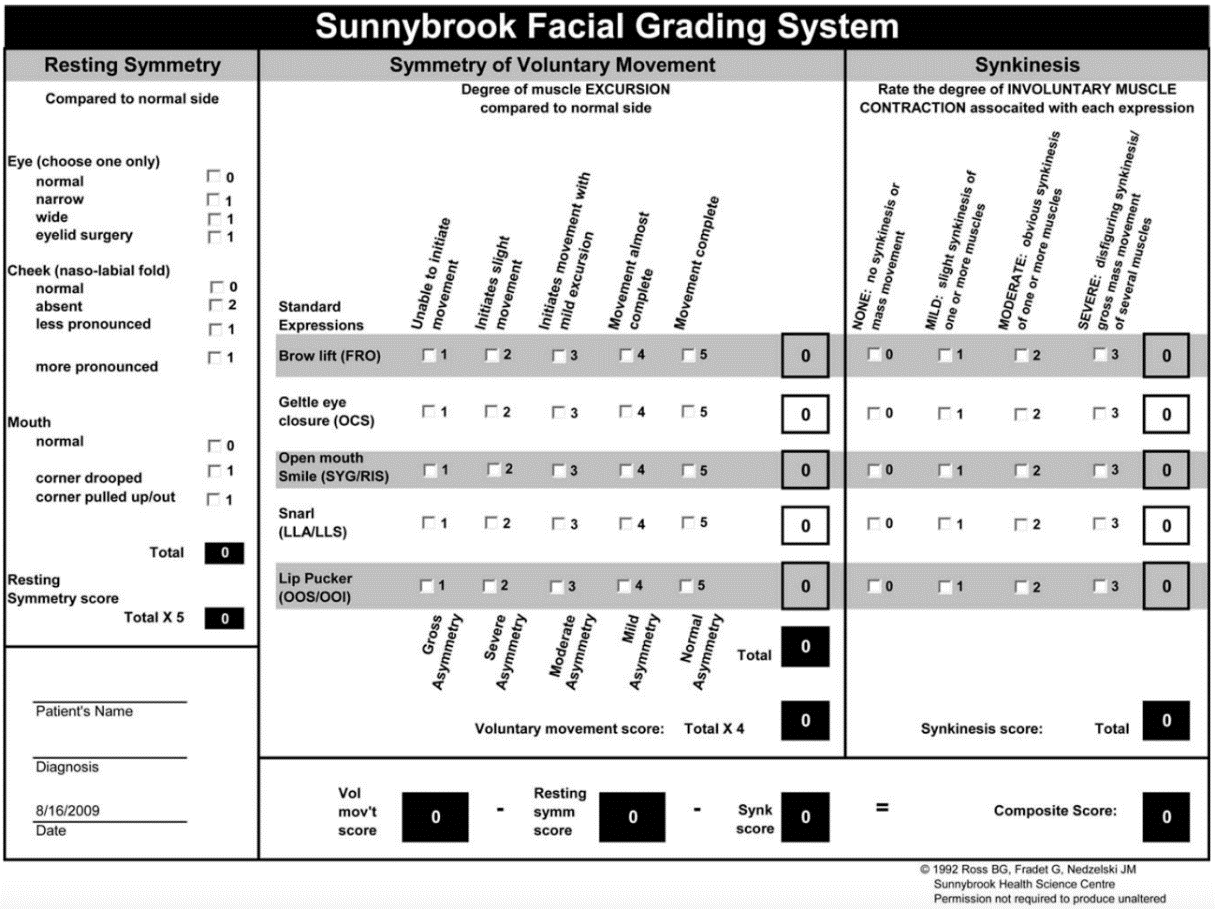


**Appendix 2** FaCE scale (KAHN, 2001).


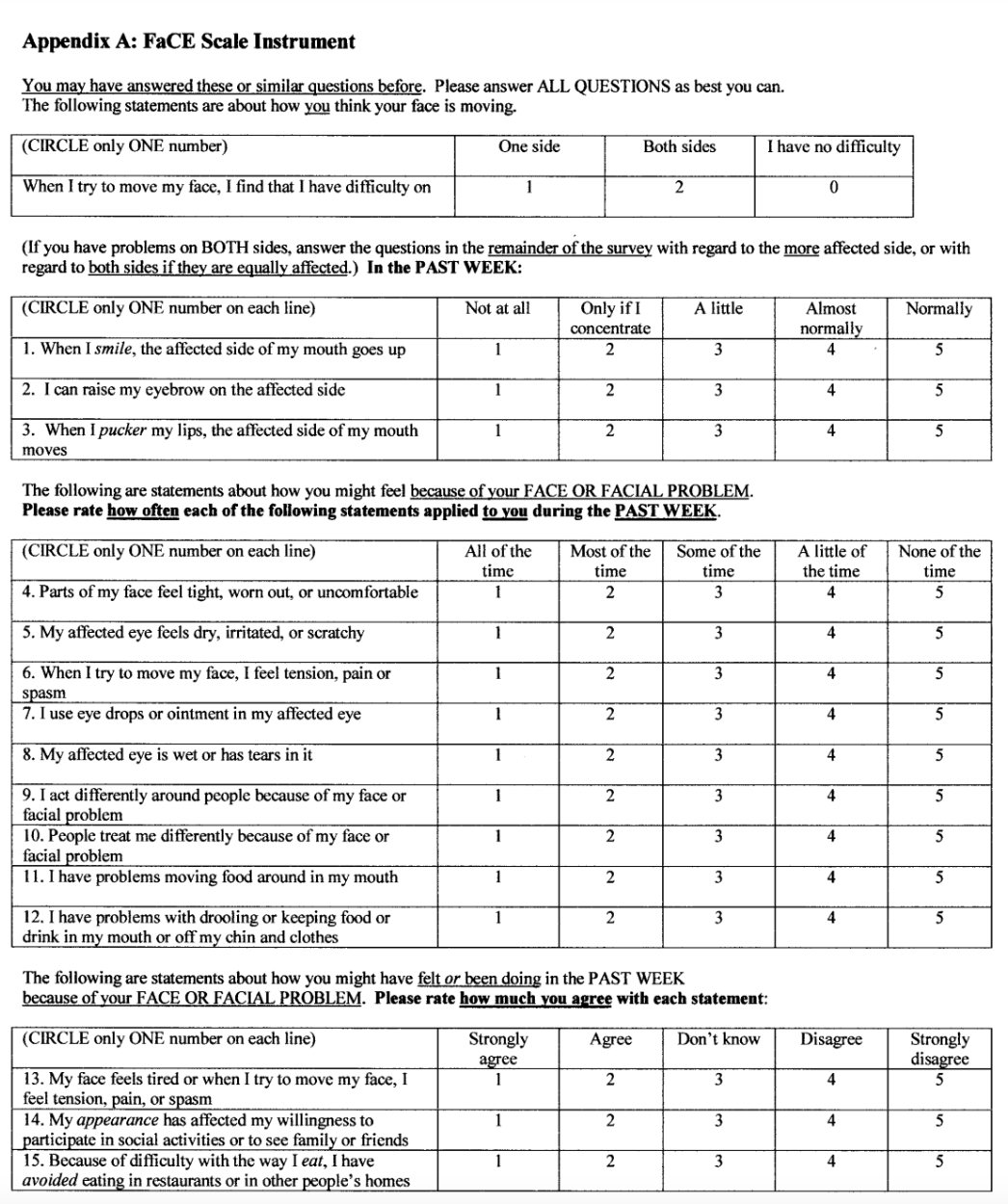


**Appendix 3** FAME scale (OJHA et al., 2022).


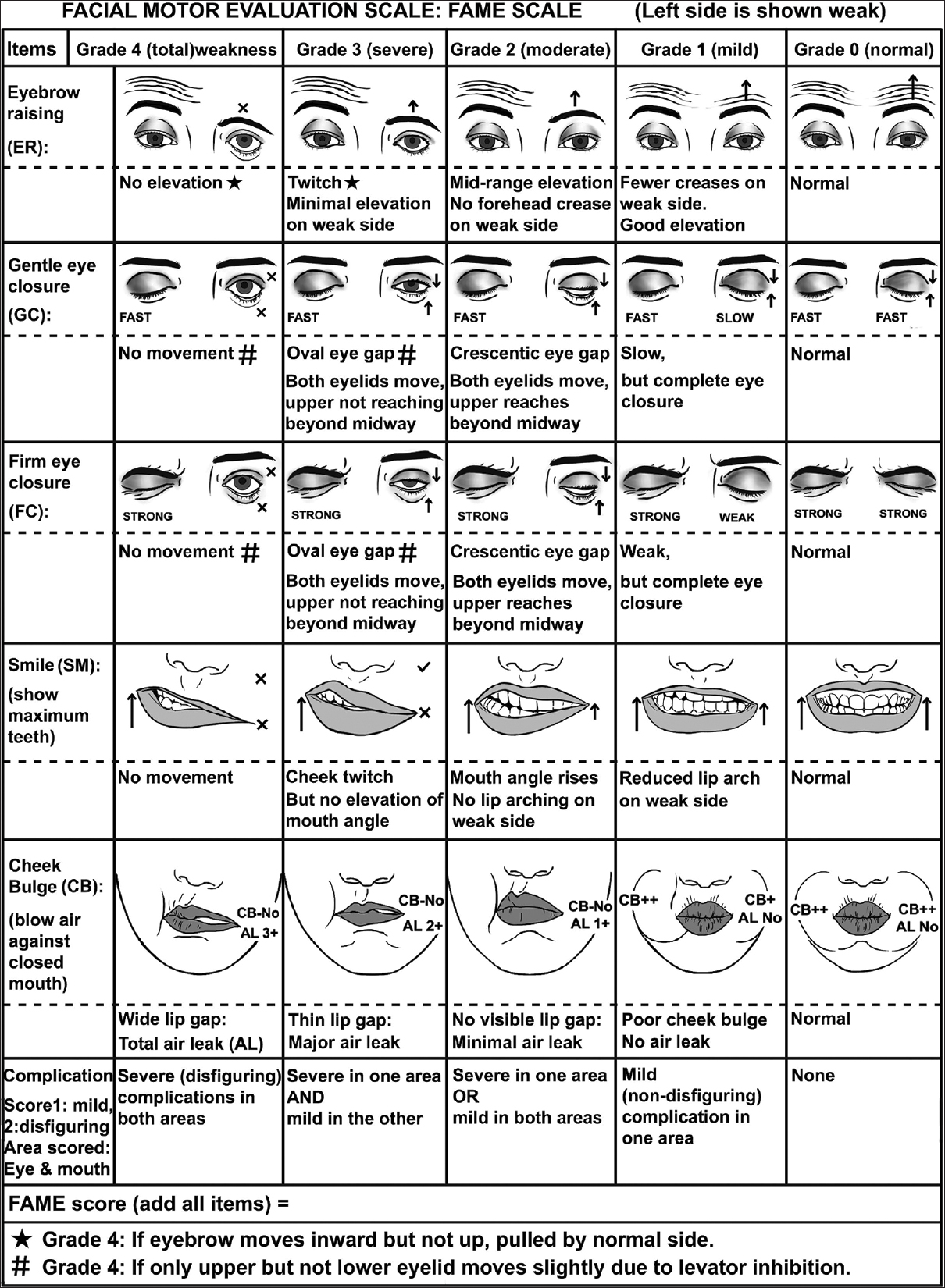

Supplement: Supplementary file 1 [file mmc1.docx]
